# Strategies to Achieve High-Performance White Organic Light-Emitting Diodes

**DOI:** 10.3390/ma10121378

**Published:** 2017-12-01

**Authors:** Lirong Zhang, Xiang-Long Li, Dongxiang Luo, Peng Xiao, Wenping Xiao, Yuhong Song, Qinshu Ang, Baiquan Liu

**Affiliations:** 1 Shunde Polytechnic, Foshan 528300, China; loezhang@foxmail.com (L.Z.); xwp556@126.com (W.X.); syhscut@vip.163.com (Y.S.); aqshu@sohu.com (Q.A.); 2 Institute of Polymer Optoelectronic Materials and Devices, State Key Laboratory of Luminescent Materials and Devices, South China University of Technology, Guangzhou 510640, China; xianglonglee@126.com; 3 School of Materials and Energy, Guangdong University of Technology, Guangzhou 510006, China; 4 School of Physics and Optoelectronic Engineering, Foshan University, Foshan 528000, China; 5 LUMINOUS! Center of Excellence for Semiconductor Lighting and Displays, School of Electrical and Electronic Engineering, Nanyang Technological University, Nanyang Avenue, Singapore 639798, Singapore

**Keywords:** white, organic light-emitting diodes, efficiency, charge, exciton

## Abstract

As one of the most promising technologies for next-generation lighting and displays, white organic light-emitting diodes (WOLEDs) have received enormous worldwide interest due to their outstanding properties, including high efficiency, bright luminance, wide viewing angle, fast switching, lower power consumption, ultralight and ultrathin characteristics, and flexibility. In this invited review, the main parameters which are used to characterize the performance of WOLEDs are introduced. Subsequently, the state-of-the-art strategies to achieve high-performance WOLEDs in recent years are summarized. Specifically, the manipulation of charges and excitons distribution in the four types of WOLEDs (fluorescent WOLEDs, phosphorescent WOLEDs, thermally activated delayed fluorescent WOLEDs, and fluorescent/phosphorescent hybrid WOLEDs) are comprehensively highlighted. Moreover, doping-free WOLEDs are described. Finally, issues and ways to further enhance the performance of WOLEDs are briefly clarified.

## 1. Introduction

Since the first organic light-emitting diode (OLED) reported by Tang et al. [1], OLEDs have received enormous interest due to their outstanding merits, including excellent efficiency, bright luminance, ultralight, ultrathin, wide viewing angle, fast switching, lower power consumption, and their compatibility with flexible substrates [2,3,4,5]. To realize next-generation solid state lighting as well as high-quality display technology, white OLEDs (WOLEDs) are urgently desired. After the pioneering work conducted by Kido et al. in 1994, the development of WOLED technology has been strikingly enhanced [6,7]. Over the past two decades, the power efficiency (PE) of WOLEDs has been improved from 0.83 lm W^−1^ to >100 lm W^−1^ [8,9,10,11,12], demonstrating the great potential of WOLEDs for applications in the lighting and display field.

With the efforts of worldwide researchers, the performance and theoretical study of WOLEDs have been gradually enhanced. For display applications, WOLEDs can be used as the backlight for flat panel displays. Furthermore, based on a white emitter with an RGB (red/green/blue) color-filter array, full-color RGBW OLED displays can be realized, which can reduce the number of manufacturing steps and eliminate the need for color patterning [13,14]. In the case of lighting applications, WOLEDs exhibit many advantages compared to conventional artificial lighting sources (e.g., candles, incandescent bulbs, and fluorescent tubes) [15,16,17]. Even compared with the widely used inorganic WLEDs, the property of less blue emission in WOLEDs renders WOLEDs able to avoid the notorious effect of blue light on melatonin suppression, which is beneficial to safeguard human health [18,19,20]. According to DisplaySearch, the market for OLED lighting applications in 2020 is predicted to reach 14 billion dollars [11]. To date, plenty of companies are pursuing WOLEDs technology, such as LG Display, Samsung, Panasonic, Konica Minolta, OLED works, OSRAM, Visionox, and so on [21]. As one example of many inspiring results, WOLEDs can simultaneously satisfy the requirement of high efficiency (133 lm W^−1^ at 1000 cd m^−2^), long lifetime (150,000 h at 1000 cd m^−2^), and high color rendering index (CRI, 84) at large-area lighting panels (100 cm^2^) [8].

To realize white emissions, the mixture of two complementary colors (i.e., blue and red, orange or yellow) or three primary colors (i.e., blue, green, and red) is generally necessary. Considering that the full wavelength at half-maximum of organic emitter is restricted (e.g., ~60 nm for 8-hydroxyquinoline aluminum (Alq_3_), ~70 nm for tris(2-phenylpyridine)iridium (Ir(ppy)_3_)), multiple emitters are usually adopted in one WOLED, which can broaden the spectral spans in the visible region (380–780 nm) [22,23,24,25,26,27,28,29,30]. Apart from the selection of excellent emitters, the careful manipulation of device engineering plays a significant role in high-performance WOLEDs. Through furthering our understanding of WOLEDs, a large number of strategies have been proposed to enhance device engineering thus far.

In this review, we first introduce the main parameters that are used to characterize the performance of WOLEDs. Then, we summarize the state-of-the-art strategies to achieve high-performance WOLEDs put forth recent years. Specifically, we comprehensively highlight the manipulation of charges and excitons distribution in the four types of WOLEDs (i.e., fluorescent WOLEDs, phosphorescent WOLEDs, TADF (thermally activated delayed fluorescent) WOLEDs, and fluorescent/phosphorescent hybrid WOLEDs). Also, we describe doping-free WOLEDs. Finally, we clarify the issues and ways to further enhance the performance of WOLEDs.

## 2. Parameters to Characterize WOLEDs

### 2.1. Emission Colors

**Figure 1:**
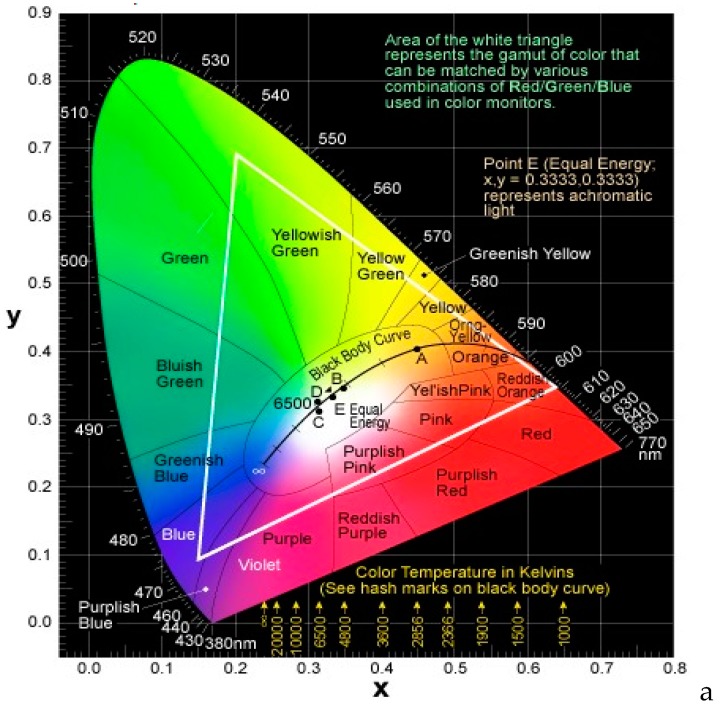
Diagram of the Commission International de L’Eclairage (CIE) chromaticity.

In general, the emission colors of WOLEDs are characterized by the Commission International de L’Eclairage (CIE) chromaticity coordinates, color stability, CRI, and correlated color temperature (CCT). In 1931, the International Commission on Illumination defined the Standard Observer for Colorimetry and the 1931 CIE System of Colorimetry. As shown in Figure 1, the vertexes of the triangle are the CIE coordinates of three primary red, green, and blue colors. In the middle area, the white light equal-energy point is (0.33, 0.33). In fact, as long as the CIE coordinates of OLEDs are located near (0.33, 0.33), the colors are considered to be white (e.g., (0.26, 0.27), (0.47, 0.43), (0.54, 0.39), (0.49, 0.41), (0.36, 0.50) and (0.39, 0.53)) [31,32,33,34,35,36].

For the color stability, it is an important parameter to evaluate the color changes of WOLEDs with the driving voltage/luminance changing [37,38]. Since WOLEDs are usually composed of multiple emitters, the recombination zone will be changed when the voltage is altered, leading to the color shift. WOLEDs usually exhibit a blue-shifted color phenomenon with the voltage increasing because the blue emitters are excited by the higher energy at higher electric field [39,40,41,42]. Moreover, due to the different lifetime of each emitter in WOLEDs, the color stability can be affected with the operating time increasing (e.g., the lifetime of the blue phosphorescent emitter iridium(III) bis[(4,6-difluorophenyl)-pyridinato-*N*,*C*^2^′] picolinate (FIrpic) is very short, <1 h) [11].

The parameter of CRI is required for lighting applications since it indicates how well lighting sources could render colors of objects they illuminate, although CRI is not critical for OLEDs in full-color displays [43]. The CRI is a measure of the color shift that an object undergoes when illuminated by the light source as compared with the color of the same object when illuminated by a reference source of comparable color temperature [44]. In other words, the CRI is the relative ability of a light source to replicate colors generated by a reference light source of the same color temperature and is determined by the light source’s spectrum. Thus, CRI is a quantitatively measurable index rather than a subjective one [45]. A reference source, such as black body radiation, is defined as having a CRI of 100 (this is why incandescent lamps have a CRI of 100, as they are almost blackbody radiators), and the test source with the same color temperature is compared against this. The values of CRI range from 0 to 100, with 100 representing no shift in color. White light sources are referenced to daylight, with incandescent lamps and natural sunlight possessing a CRI of 100, tungsten halogen lamps near 95, fluorescent bulbs having ratings between 60 and 99, mercury lamps near 50, high-pressure sodium lamps about 20, and low-pressure sodium vapor lamps, which are monochromatic, have a CRI that is nearly 0. For a source to be human eye-friendly, a WOLED with CRI ≥80 is needed [46]. Since D’Andrade et al. took the first step to demonstrate that the CRI of WOLEDs could be greatly enhanced from 50 to 83 in 2002 [47], a great deal of attention has been dedicated to the improvement of CRI, which is expected to meet commercial requirements. In recent years, in order to satisfy the demand of high-quality lighting systems, such as those used for surgery, photography, and exhibitions of museums, WOLEDs with very-high CRI (≥90) have become extremely desirable [48]. As a matter of fact, some efforts have been made in the pursuit of very high-CRI WOLEDs [49,50,51,52,53,54,55,56].

CCT, measured in degrees Kelvin (K), is a specification of the absolute temperature of a blackbody whose chromaticity most nearly resembles that of the light source. To distinguish WOLEDs, warm light is usually about 2700 K, moving to neutral white at about 4000 K, and to cool white at 5000 K or more. As a matter of fact, warm-white and cool-white WOLEDs have been extensively reported [57,58,59,60,61]. Furthermore, to mimic sunlight, WOLEDs should exhibit a wide CCT span (2500–8000 K), since the sun possesses a time-changing CCT at different regions and times on the earth (e.g., 2500 K at sunset, 3250 K at sunrise, and 5500 K at noon or 8000 K at noon in high-latitude countries) [20].

### 2.2. Emission Efficiency

In OLEDs, the emission efficiency can be analyzed by the EQE (external quantum efficiency), CE (current efficiency), and PE. For EQE (*η*_ext_), it is expressed as [23]:

(1) $$\eta_{\mathrm{ext}}=\eta_{out}\cdot r\cdot q\cdot \gamma$$

where *η_out_* is the outcoupling factor, *r* is the fraction of excitons that can potentially radiatively decay, *q* is the photoluminescence quantum efficiency (PLQY) of emitters, and *γ* is the charge balance (*γ* ≤ 1). If emitters are set, the *r* and *q* will be decided. Moreover, the electronic procedure occurring inside the device usually has no influence on *η_out_*. Thus, from the perspective of device engineering, the *η*_ext_ is mostly sensitive to *γ*. On the other hand, the CE ($\eta_{L}$) is defined as [11]:

(2) $$\eta_{L}=L/J=(LS)/I$$

where *L* is the luminance, *S* is the emission area, and *I* is the current. Moreover, assuming the emission pattern is the Lambertian type, the PE ($\eta_{P}$) is described as [11]:

(3) $$\eta_{P}=(\pi \eta_{L})/U$$

where *U* is the driving voltage. The connection between EQE and PE can be expressed as:

(4) $$\eta_{P}\propto \frac{\eta_{\mathrm{ext}}}{U}$$

Simply, PE is expressed as:

(5) $$\eta_{P}\propto \frac{\gamma }{U}$$

Therefore, to achieve high PE, *γ* should be high while *U* should be low. Due to the unfavorable energy barriers between different layers together with the fact that electrons are minor charges in organic materials, charges balance and transport are usually unsatisfactory [62,63,64,65,66]. Therefore, charges should be not only well balanced but also effectively transported.

### 2.3. Lifetime

The lifetime of OLEDs usually means the time when the luminance decays to its half value. Kodak company first proposed an equation to describe the lifetime [67]:

(6) $$L_{0}\times t_{1/2}=C$$

where *L*_0_ is the initial luminance, $t_{1/2}$ is the half-life and *C* is a constant. Although this equation is not very accurate and many different equations have been proposed to characterize the lifetime, these reported equations are still somewhat similar since they have the same conclusion that the higher initial luminance will result in a shorter half-life. Currently, the most widely used and relatively accurate equation is [68,69,70]:

(7) $${L_{0}}^{n}\times t_{1/2}=C$$

where *n* is the acceleration coefficient. *n* depends on the materials set, device architectures, and emission colors [71,72,73]. For practical uses, WOLEDs need to possess a long lifetime ≥10,000 h at a high brightness ≥1000 cd/m^2^ as well as the fluorescent lamp efficiency (40–70 lm W^−1^). So far, although a large number of companies have claimed that they are able to develop long-lifetime and high-efficiency WOLEDs, detailed information of such products has not been released. In the published literature, long-lifetime WOLEDs are usually achieved by using conventional fluorescent emitters, while no long-lifetime phosphorescent WOLED has been documented since there are no stable blue phosphorescent emitters to date [74,75].

## 3. Fluorescent WOLEDs

### 3.1. Fluorescence and Phosphorescence

**Figure 2:**
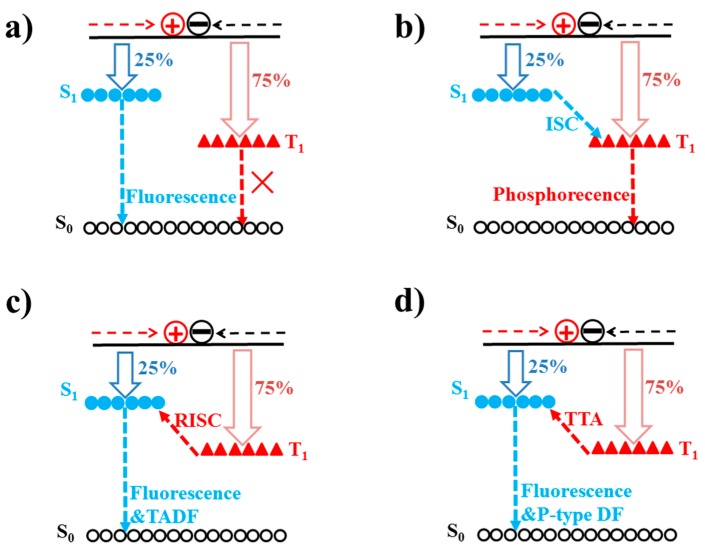
Exciton energy diagram and the possible decay ways of singlet and triplet excitons. T_1_, S_1_ and S_0_ represent the triplet energy, singlet energy, and ground state, respectively. (**a**) Fluorescence in conventional fluorescent emitters; (**b**) Phosphorecence in phosphorescent emitters; (**c**) Fluorescence and (thermally activated delayed fluorescence) TADF emitters; (**d**) Fluorescence and p-type delayed fluorescence in triplet-triplet annihilation (TTA) based emitters.

Based on adopted emitters, four types of WOLEDs have been usually reported, such as fluorescent, phosphorescent, TADF, and hybrid WOLEDs. Due to the effect of spin statistics, when holes injected from the anode meet electrons injected from the cathode, singlet and triplet excitons will be formed with a ratio of 1:3 [76,77,78]. For conventional fluorescent emitters, only the singlet excitons (25%) can emit light since the radiative decay of triplet excitons (75%) is spin forbidden, as shown in Figure 2a. For phosphorescent emitters, they can not only harvest triplet excitons via the triplet-triplet energy transfer, but can also harvest singlet excitons via the singlet-triplet intersystem crossing process (ISC) due to the heavy-atom effect, leading to a maximum internal quantum efficiency (IQE) of 100% [79,80,81], as shown in Figure 2a. In TADF emitters, triplet excitons can be harvested as delayed fluorescence through their up-conversion from a lowest triplet state to the lowest singlet state by inducing efficient reverse intersystem crossing (RISC) [82,83,84], as shown in Figure 2c. The molecular design of TADF materials requires a small energy gap between the lowest triplet state and lowest singlet state, typically less than 0.2 eV, to enhance thermal up-conversion [85]. On the other hand, triplet-triplet annihilation (TTA, also known as triplet fusion) is important as it makes dark triplet states accessible for the emission of p-type DF [86,87,88,89], as shown in Figure 2d. TTA is a bimolecular process that occurs between two molecules in their lowest triplet excited state, which form one higher excited singlet, triplet, or quintet state. If the formed excited state is a singlet, the TTA process can result in p-type DF (a minimum of two triplet states to produce one singlet excited state). In a word, the maximum theoretical IQE of fluorescent, phosphorescent, TADF, and TTA emitters are 25%, 100%, 100%, and 62.5% (25% + 75%/2), respectively.

### 3.2. Device Architectures

Despite their different molecular weights, both polymer and small-molecule organic materials can be used to develop WOLEDs. The solution-processed technique is required for polymer WOLEDs [90,91,92,93], while the solution-processed and vacuum evaporation techniques can be applied to small-molecule WOLEDs [94,95,96]. Herein, we mainly focused on small-molecule WOLEDs.

**Figure 3:**
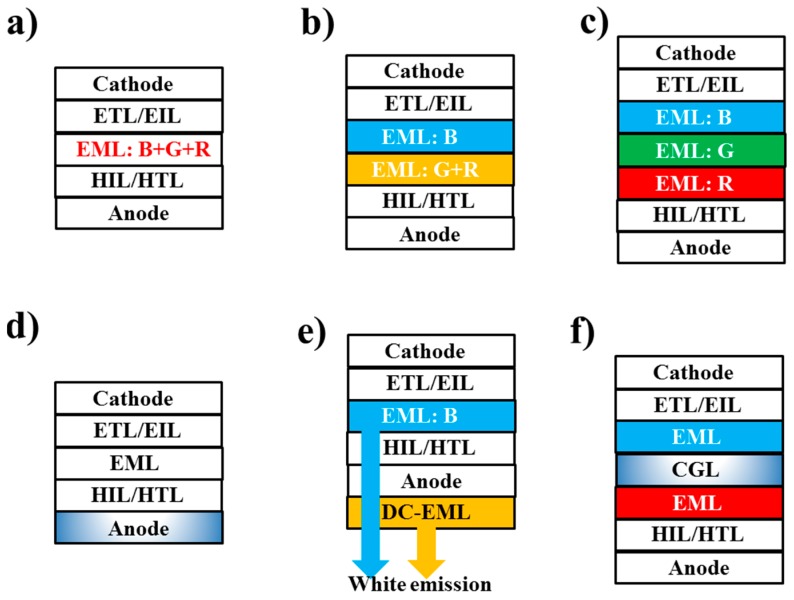
Schematic diagram of device architectures of WOLEDs. HIL is the hole injection layer, HTL is the hole transport layer, ETL is the electron transport layer, EIL is the electron injection layer, DC-EML is the down-conversation EML, B is blue, G is green, and R is red. (**a**) Single-EML WOLEDs; (**b**) Double-EML WOLEDs; (**c**) Triple-EML WOLEDs; (**d**) Microcavity WOLEDs; (**e**) Down-conversation WOLEDs; (**f**) Tandem WOLEDs.

So far, a large number of device architectures have been designed to fabricate WOLEDs. According to the number of emitting layers (EMLs), the device architectures can be simply classified into two kinds, namely single-EML and multi-EML WOLEDs, as shown in Figure 3a–c. For single-EML WOLEDs, two methods are usually adopted to generate the white emissions [97,98,99]. On one hand, by managing the incomplete energy transfer from hosts to guests in EMLs, the host materials can also function as the blue emitters to produce blue emissions, in which the concentration of guests is generally low (e.g., <1%) to ensure the white emissions. On the other hand, the energy transfer from hosts to guests is complete only where various-color guests are combined to generate the white emissions. For multi-EML WOLEDs, their structures, fabrication procedures, and device engineering methods are more complicated than single-EML WOLEDs. However, their performance is usually more satisfactory, such as low turn on voltage, high efficiency, and desired white CIE coordinates. Generally, all of these structures can be used to develop any type of WOLED. Therefore, the careful manipulation of charges and excitons distribution in WOLEDs is key to the high performance.

For other structures (e.g., microcavity WOLEDs, down-conversation WOLEDs, and tandem WOLEDs), attention is mainly devoted to other aspects besides the manipulation of charges and excitons distribution. For example, the main issue for microcavity WOLEDs (Figure 3d) is how to avoid the narrow and angular dependent emission characteristics, since the commonly used thin metal layers as the electrodes play a detrimental role in these two factors [100,101,102,103,104,105]. For down-conversation WOLEDs (Figure 3e), the main issue is how to develop an efficient blue OLED, since the down-conversation layer only can be excited by absorbing the blue emission [106,107,108,109]. For tandem WOLEDs (Figure 3f), the main focus is how to design an effective charge generation layer (CGL), since the CGL plays a significant role in ensuring high efficiency and long lifetime [110,111,112,113,114,115,116,117,118,119]. Based on these facts, we would like to only briefly introduce these structures in this review.

### 3.3. High-Efficiency Fluorescent WOLEDs

**Figure 4:**
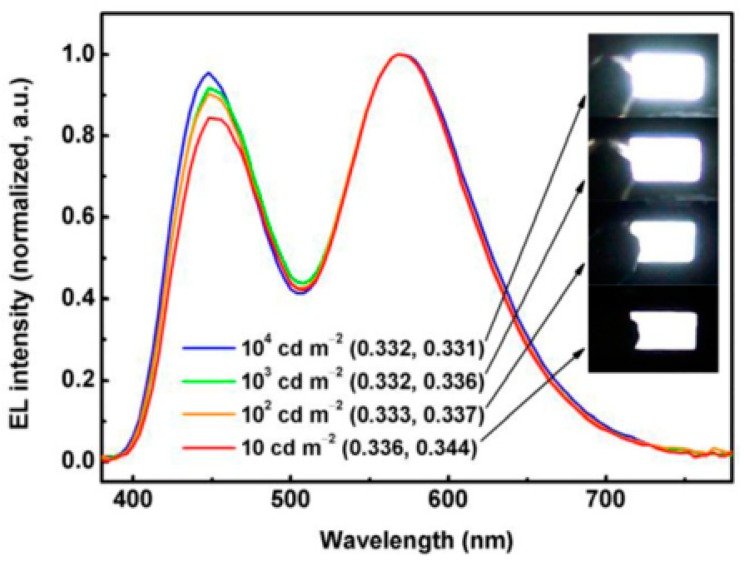
Spectra of the WOLED at various luminances. Reproduced from Reference [120].

Although the theoretical maximum EQE for conventional fluorescent emitters is 5%, there are some strategies that can break this limitation in fluorescent WOLEDs, achieving high efficiency. A representative strategy was proposed by Yang et al. for their device, in which they employed a deep blue-emitting beryllium complex bis(2-(2-hydroxyphenyl)-pyridine)beryllium (Bepp_2_) doped with a wide-bandwidth orange-emitting fluorescent dye 4-(dicyanomethylene)-2-methyl-6-(4-dimethylaminostyryl)-4-*H*-pyran (DCM) through incomplete energy transfer from the blue host to the orange dopant [120]. They first fabricated a single-EML WOLED with the structure of ITO/4,4-bis(*N*-(1-naphthyl)-*N*-phenylamino)biphenyl (NPB, 400 nm)/Bepp_2_: DCM (0.5%, 45 nm)/LiF/Al. However, with an increase in blue-emission intensity, brightness-dependent electroluminescent (EL) spectra became more obvious than the orange dopant as the brightness increased. Since the EL of this device originated from the uniformly doped layer, the relative change in EL intensity for blue and orange emissions was partly attributed to the requirement of higher energy excitation for blue light. Besides, the competition between efficient energy transfer from the blue host to the orange dopant and exciton formation on the orange dopant (DCM) by charge trapping would have a considerable effect on the relative emission intensity. To achieve a WOLED with stable colors, a device was fabricated and designed by decreasing the doping concentration from 0.5 to 0.2 wt % within 3 nm of EML adjacent to the HTL/EML interface and by maintaining the total thickness of the doped EML at 45 nm. The structure is ITO/NPB (400 nm)/Bepp_2_: DCM (0.2 wt %, 3 nm)/Bepp_2_: DCM (0.5 wt %, 42 nm)/LiF/Al. Within the luminance of 10–10,000 cd/m^2^, the device shows a very pure-white and stable color with the CIE coordinates of (0.334 ± 0.002, 0.337 ± 0.007), close to the white-equivalent point of (0.333, 0.333), as shown in Figure 4. The CRI is as high as 79–81, with a CCT between 5400 and 5600 K. Moreover, the device gave the maximum forward-viewing EQE and PE of 5.6% and 9.2 lm W^−1^, respectively, thus ranking among the best fluorescent WOLEDs fabricated to date.

### 3.4. Long-Lifetime Fluorescent WOLEDs

**Figure 5:**
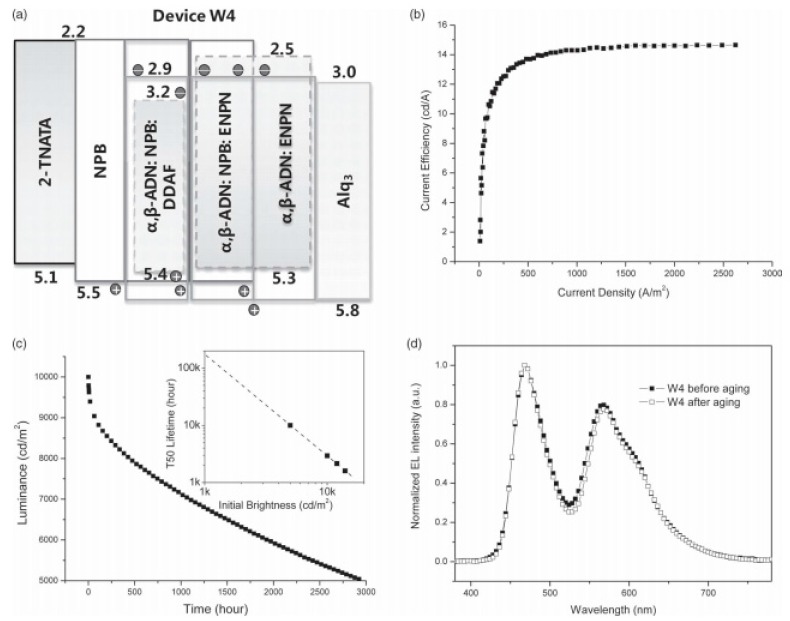
Characteristics of the optimized WOLED: (**a**) device structure; (**b**) CE versus density curve; (**c**) luminance decay curve; the inset shows the lifetime versus initial luminance relationship; (**d**) EL spectra before and after accelerated aging at 10,000 cd m^−2^. Reproduced from Reference [123].

Despite the fact that fluorescent WOLEDs exhibit low efficiencies [121,122], long-lifetime devices are usually reported by fluorescent structures due to the stable fluorophorees, particularly for blue fluorophorees. In fact, the lifetime of organic devices remains a major challenge that must be overcome prior to the wide application of WOLEDs technology. In 2011, Duan et al. presented a strategy to achieve WOLEDs with an extremely long lifetime by the wise control of the recombination zone [123]. In their device, a blue EML of 6,6′-(1,2-ethenediyl)bis(*N*-2-naphthalenyl-*N*phenyl-2-naphthalenamine (ENPN) doped 9-(1-naphthyl)-10-(2-naphthyl)-anthracene (α,β-ADN) was deposited on top of the mixed host blue EML to prevent hole penetration into the ETL and to attain better confinement of carrier recombination. Therefore, the utilization of double blue EMLs can stabilize the blue emission, which is the key feature to their device. Figure 5a depicts the structure: ITO/2-TNATA 100 nm/NPB 20 nm/78% α,β-ADN 20% NPB 2% 3,11-Diphenylamino-7,14-diphenylacenaphtho[1,2-k]fluoranthene (DDAF) 10 nm/78% α,β-ADN: 20% NPB: 2% ENPN10 nm/98% α,β-ADN: 2% ENPN 15 nm/Alq_3_ 20 nm/LiF/Al, where the 10 nm mixed-host blue EML (78% α,β-ADN: 20% NPB: 2% ENPN) was utilized to broaden the recombination zone and dilute the concentration of any degradation related quenching species, the second 15 nm blue EML (98% α,β-ADN: 2% ENPN) was then deposited onto the mixed-host blue EML to achieve better charge confinement, and a mixed-host yellow EML (78% α,β-ADN 20% NPB 2% DDAF) was combined to furnish the white emission. As a result, the WOLED showed a maximum CE of 14.7 cd A^−1^ (Figure 5b). Remarkably, a lifetime of over 150,000 h at an initial brightness of 1000 cd m^−2^ was obtained (Figure 5c), which is the longest value among WOLEDs in the published literature so far. Furthermore, the EL spectra of the WOLED showed almost no color-shifting after accelerated aging (Figure 5d).

## 4. Phosphorescent WOLEDs

### 4.1. Single-EML Phosphorescent WOLEDs

#### 4.1.1. Single-EML Phosphorescent WOLEDs with Unipolar Hosts

Similar to fluorescent WOLEDs, phosphorescent WOLEDs can also be generally classified into single-EML and multi-EML phosphorescent WOLEDs. However, unlike single-EML fluorescent WOLEDs, there is usually only one approach to realize single-EML phosphorescent WOLEDs, namely using various-color guests to generate the white emissions (the energy transfer from hosts to guests is complete).

**Figure 6:**
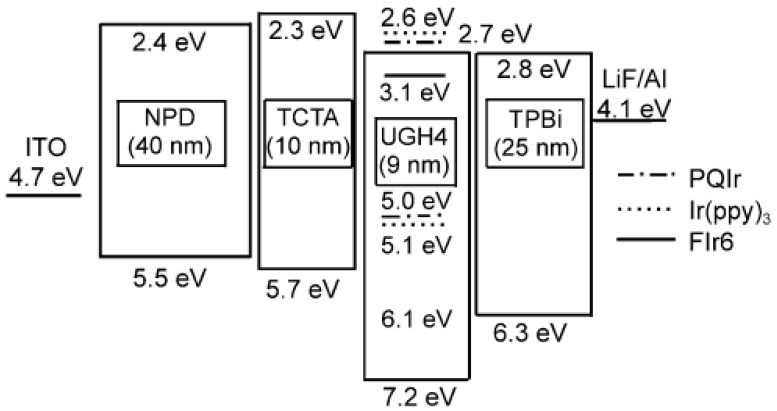
Structure of the WOLED and the proposed energy level. NPD is 4,4′-bis[*N*-(1-naphthyl)-*N*-phenyl-amino]biphenyl. Reproduced from Reference [124].

A strategy to achieve single-EML phosphorescent WOLEDs is using n-type hosts doped with various-color guests. For example, D’Andrade et al. co-doped bis(4′,6′-difluorophenylpyridinato) tetrakis(1-pyrazolyl)borate (FIr6, blue phosphor), Ir(ppy)_3_ (green phosphor), and iridium(III) bis(2-phenyl quinoly-*N*,*C*^2^′) acetylacetonate (PQIr, red phosphor) into the n-type host p-bis(triphenylsilyly)benzene (UGH2) with a wide energy gap, developing a triple-doped single-EML WOLED, as shown in Figure 6 [124]. The WOLED can exhibit a maximum total PE of 42 lm W^−1^, which was the highest among WOLEDs at that time. The high performance can be attributed to: (1) the use of a thin EML (9 nm), which can reduce the voltage, enhancing the PE; (2) 4,4′,4′′-tri(9-carbazoyl) triphenylamine (TCTA) and 2,2′,2′′-(1,3,5-benzinetriyl)-tris(1-phenyl-1-*H*-benzimidazole) (TPBi) functioning as the HTL and ETL, respectively, forming a charge/exciton structure. This is because the lowest unoccupied molecular orbital (LUMO) of TCTA is ≥0.3 eV higher than that of the host and guests in the EML, while the highest occupied molecular orbital (HOMO) of TPBi is 0.2 eV higher than FIr6. Also, the energy gap of TCTA and TPBi are 3.4 and 3.5 eV, respectively, which are much higher than those of FIr6. Thus, the combined TCTA and TPBi effectively prevent the charges and excitons transporting from the EML to HTL or ETL. Finally, (3) charges can be directly injected into FIr6 and then formed excitons, eliminating the energy transfer from the host UGH2 to the guest FIr6, which can reduce the loss of exchange energy in the host-guest energy transfer process.

**Figure 7:**
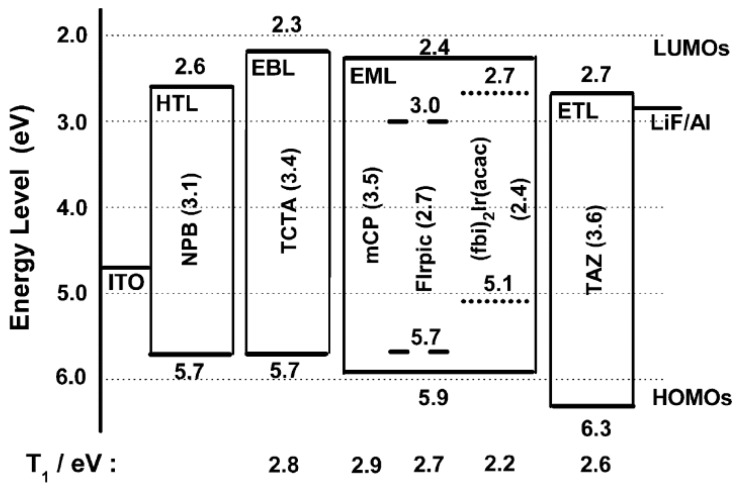
Structure of the WOLED and the proposed energy level. TAZ is 3-(4-biphenyl)-4-phenyl-5-(4-tert-butylphenyl)-1,2,4-triazole, which was employed as the ETL. Reproduced from Reference [125].

Another strategy to achieve single-EML phosphorescent WOLEDs is using p-type hosts doped with various-color guests. Wang et al. incorporated FIrpic (blue phosphor) and bis(2-(9,9-diethyl-9*H*-fluoren-2-yl)-1-phenyl-1*H* benzoimidazole-*N*,*C*^3^)iridium(acetylacetonate) [(fbi)_2_Ir(acac), orange phosphor] into the p-type host 1,3-bis(9-carbazolyl)benzene (mCP), constructing a high-efficiency single-EML WOLED, as shown in Figure 7 [125]. The device could exhibit a maximum total PE of 72.2 lm W^−1^, which was the highest among single-EML WOLEDs at that time. At 500 cd m^−2^, the total efficiency qA 32.3 lm W^−1^. In addition, a CRI of 71 could be obtained at 13 V. The key feature is the careful manipulation of two exciton-formation modes, namely host-guest energy transfer for FIrpic and direct exciton formation for (fbi)_2_Ir(acac), within an energetic, well-like, single-emissive region. The energy on mCP can be easily transferred to FIrpic, reducing the unfavorable energy loss by the mCP emission. Particularly, the multifunctional orange dopant (fbi)_2_Ir(acac) (serving as either hole-trapping site or electron-transporting channel) is essential to this concept, as it can make an improved charge balance and broaden the recombination zone in the p-type host mCP-based EML. Thus, there are two parallel pathways to channel the overall excitons to both dopants within the EML, leading to an improved charge balance and further reduction of the unfavorable energy losses. Hence, an extremely high-efficiency WOLED with nearly 100% IQE was realized. Subsequently, they combined this efficient WOLED as the single unit and 2,9-dimethyl-4,7-diphenyl-1,10-phenanthroline (BCP): Li/MoO_3_ as the CGL, developing a high-efficiency tandem WOLED with a maximum PE of 45.2 lm W^−1^ and a CE of 110.9 cd A^−1^ [126].

#### 4.1.2. Single-EML Phosphorescent WOLEDs with Bipolar Hosts

Although single-EML phosphorescent WOLEDs with unipolar hosts have been demonstrated to show high efficiency, poor color-stability and serious efficiency roll-off are commonly difficult to avoid [127,128,129,130]. The unsatisfactory performance can be attributed to the intrinsic quality of unipolar hosts, since they usually only transport holes or electrons, resulting in charge accumulation/recombination occurring close to the EML/ETL or HTL/EML interface. Thus, narrow recombination zones and then TTA, triplet-polaron quenching (TPQ), and exciton diffusion may occur, which would be detrimental to the performance.

**Figure 8:**
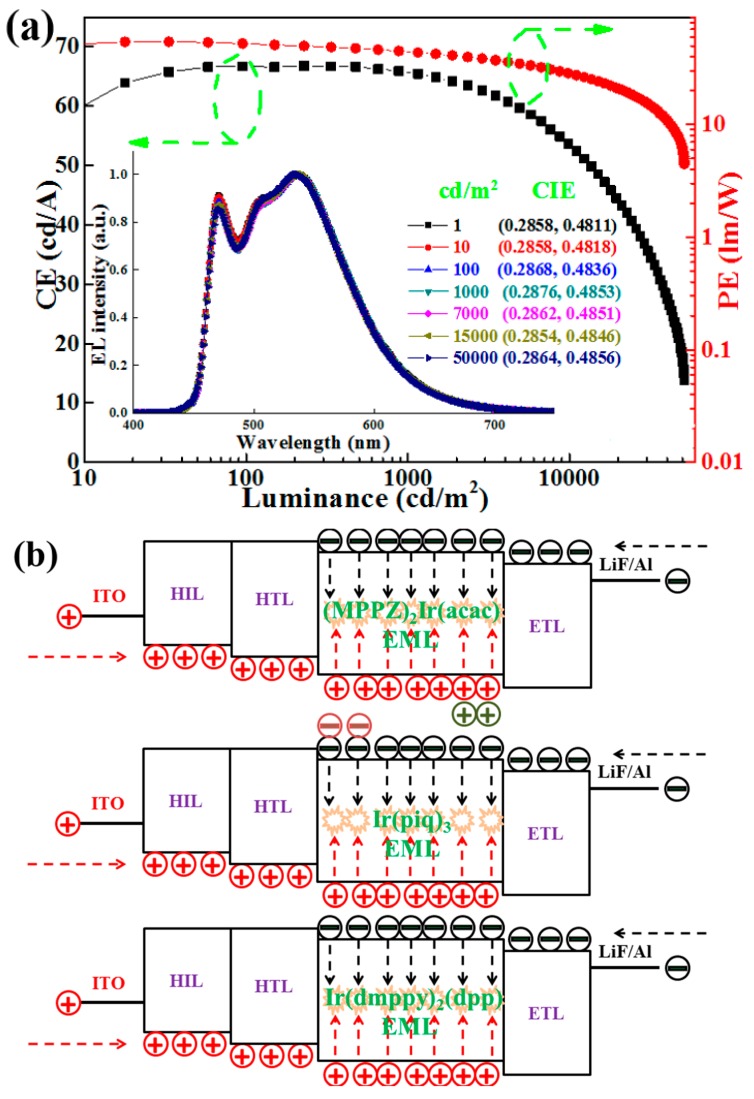
(**a**) Forward-viewing CE and PE. Inset: EL spectra; (**b**) Diagrams of EL processes in WOLEDs with the guests of (MPPZ)_2_Ir(acac), Ir(piq)_3_ and Ir(dmppy)_2_(dpp). Reproduced from Reference [132] with permission from the Royal Society of Chemistry.

To solve these issues, the introduction of single-EML WOLEDs employing bipolar hosts is one of the most ideal strategies [131]. As bipolar materials facilitate both hole and electron transport, more balanced charges and broader recombination regions within the EMLs can be guaranteed, resulting in devices with the potential of higher efficiency, lower efficiency roll-off, and more stable color. For example, Liu et al. built a single-EML WOLED by co-doping FIrpic and bis(2-phenyl-4,5-dimethylpyridinato)[2-(biphenyl-3-yl)pyridinato] iridium(III) [(Ir(dmppy)_2_(dpp)] into 2,6-bis(3-(carbazol-9-yl)phenyl)pyridine (26DCzPPy), simultaneously achieving simplicity, extreme efficiency, reduced efficiency roll-off, and excellent color-stability [132]. The configuration of their WOLED was as follows: ITO/MeO-TPD (*N*,*N*,*N*′,*N*′-tetrakis(4-methoxyphenyl)-benzidine): F4-TCNQ (tetrafluoro-tetracyanoqino dimethane) (100 nm, 4%)/TAPC (1-bis[4-[*N*,*N*-di(4-tolyl)amino]phenyl]-cyclohexane, 20 nm)/26DCzPPy: FIrpic: Ir(dmppy)_2_(dpp) (11 nm, 1: 22.5%: 1.1%)/TmPyPB (1,3,5-tri(*m*-pyrid-3-yl-phenyl)benzene, 50 nm)/LiF/Al. The device exhibited total efficiencies of 111.7 cd A^−1^ and 75.5 lm W^−1^ at 1000 cd m^−2^, as shown in Figure 8a. Besides, a maximum CE and PE of 113.6 cd A^−1^ and 92.5 lm W^−1^ were obtained, respectively, only slightly decreasing to 101.3 cd A^−1^ and 58.8 lm W^−1^ even at 5000 cd m^−2^, indicating a low efficiency roll-off. Additionally, the color variation in the whole range of luminances was (0.00, 0.00), thus representing the first single-EML WOLED with an extremely stable color (Figure 8a inset). Factors for the high performance were discussed: (1) Two-complementary color WOLEDs can show higher efficiency compared with three-color WOLEDs; (2) The bipolar host 26DCzPPy plays a vital role due to its high T_1_ (2.71 eV), similar hole and electron mobility (~10^−5^ cm^2^/(V s)), and suitable HOMO and LUMO; (3) A carrier/exciton-confining structure was used; (4) The appropriate thickness of EML (11 nm) was optimized; (5) Both hole and electron transport was greatly reduced due to the two multifunctional guests, resulting in the recombination ratio remaining unchanged with the raising luminance, which is beneficial to obtain stable colors. This is because the charge transport can be slowed down by the host-guest energy level discrepancy in the EML, and the recombination ratio can be constant, greatly stabilizing the color (Figure 8b). By replacing Ir(dmppy)_2_(dpp) with iridium(III) diazine complexes [(MPPZ)_2_Ir(acac)], which has a HOMO of 5.31 eV and a LUMO of 3.13 eV, the device showed an unstable color. This is because (MPPZ)_2_Ir(acac) cannot lower the hole mobility due to a low concentration (1.1%) together with the fact that (MPPZ)_2_Ir(acac) has a similar HOMO with TAPC. Besides, by replacing Ir(dmppy)_2_(dpp) with tris(1-phenylisoquinolinolato-*C*^2^,*N*) iridium(III) [Ir(piq)_3_], which has a HOMO of 5.0 eV and a LUMO of 2.7 eV, an unstable color was obtained. This is because Ir(piq)_3_ cannot lower the electron mobility owing to the low concentration (1.1%) and Ir(piq)_3_ has a similar LUMO with TmPyPB. Therefore, the harness of charges and excitons distribution by using multifunctional guests is crucial for stabilizing the color.

#### 4.1.3. Single-EML Phosphorescent WOLEDs with Solely Exciplex Hosts

Apart from the abovementioned unipolar or bipolar hosts, the mixture of p-type and n-type materials to form exciplex hosts is also an effective strategy to demonstrate high-performance single-EML phosphorescent WOLEDs. This is because the charge injection barrier issue can be nearly eliminated and give an efficient TADF up-conversion due to the intermolecular donor-acceptor construction and sufficiently small S_1_-T_1_ splitting (<0.1 eV). Furthermore, the relatively high T_1_ of exciplex will reduce the energy back-transfer (i.e., from T_1, guest_ to T_1, host_) rate constant, preventing the exciton leakage [12]. Hence, exciplex hosts can possibly make a tradeoff among the 100% IQE, low voltage, and good exciton confinement in the EML.

**Figure 9:**
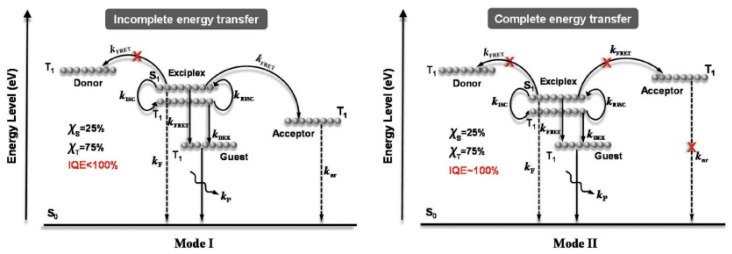
Two different energy-transfer modes. Donor and acceptor are the constituting molecules for exciplex. S_1_ and T_1_ are the singlet and triplet states, respectively. k_FRET_, k_DEX_, k_ISC_, k_RISC_, k_F_, and k_P_ are the rate constant of Förster energy transfer, Dexter energy transfer, ISC, RISC, fluorescence and phosphorescence processes, respectively. Reproduced from Reference [12].

Wu et al. reported a single-EML WOLED by using donor mCP and acceptor 4,6-bis[3,5-(dipyrid-4-yl)phenyl]-2-methylpyrimidine (B4PyMPM) as the solely exciplex host, obtaining a forward-viewing PE of 105.0 lm W^−1^, CE of 83.6 cd A^−1^, and EQE of 28.1% [12]. The configuration for this WOLED is as follows: ITO (110 nm)/TAPC (40 nm)/TCTA (10 nm)/mCP (10 nm)/mCP: 50 wt % B4PyMPM: 15 wt % FIrpic: 0.2 wt % (acetylacetonato)bis[2-(thieno[3,2-c]pyridin-4-yl)phenyl]iridium(III) (PO-01) (20 nm)/B4PyMPM (50 nm)/Liq (0.8 nm)/Al (120 nm). The high performance can be explained as follows: (1) The exciplex was exclusively employed as the host and at the same time co-doped it with two-color phosphors, prohibiting the shortcomings of low efficiency and pronounced efficiency roll-off; (2) By using energy level matching HTL and ETL, the structural heterogeneity was reduced. Moreover, high hole carrier mobility of mCP (5 × 10^−4^ cm^2^ V^−1^ s^−1^) and electron mobility of B4PyMPM (1 × 10^−4^ cm^2^ V^−1^ s^−1^) were used. Hence, low voltages were realized (e.g., 1 cd m^−2^ at 2.5 V); (3) The perfectly confined excitons fully eliminated the exciton leakage from the emission zone, enhancing the efficacy. As shown in Figure 9 left, if acceptor molecules with low T_1_ were employed, some of the excitons from the exciton formation zone would be trapped by the acceptor (transfer mode I). Hence, excitons transferred to the acceptor molecules would decay via a nonradiative path owing to their rather low photoluminescence quantum yields (<20%), thereby causing undesirable exciton leakage and accordingly low efficiency. As shown in Figure 9 right, if acceptor molecules with high T_1_ were employed, the leakage phenomenon would be suppressed (transfer mode II). Hence, all excitons are transferred only to the emitter molecules via either long-range dipole-dipole coupling (Förster energy transfer) or short-range exchange interaction (Dexter energy transfer). Thus, the emitter molecules can utilize all the excitons and consequently deliver a 100% IQE.

### 4.2. Multi-EML Phosphorescent WOLEDs

#### 4.2.1. Carrier- and Exciton-Confining Structure

**Figure 10:**
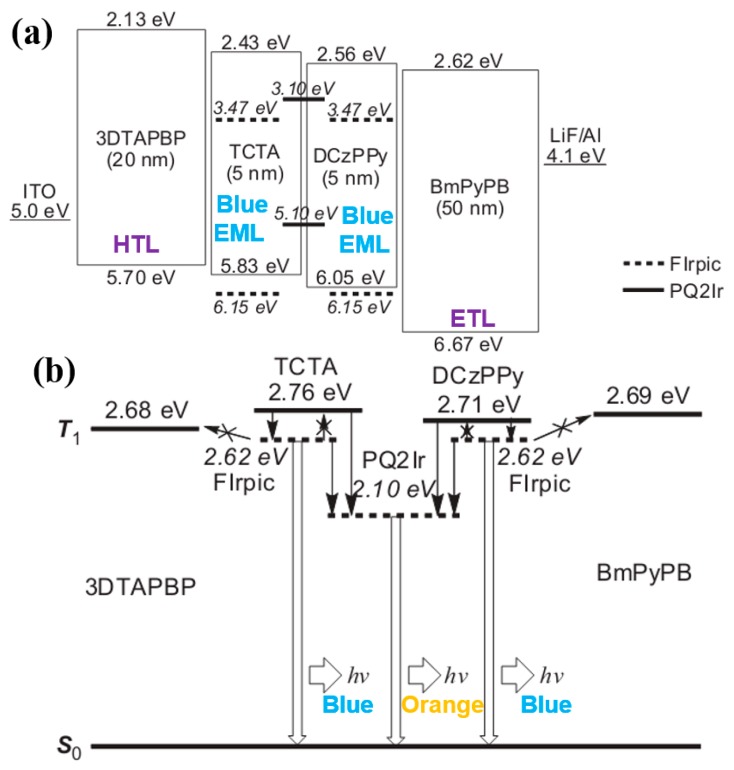
(**a**) The device structure of the WOLED and the proposed energy level; (**b**) Diagrams of EL processes in WOLEDs. Reproduced from Reference [133].

For multi-EML phosphorescent WOLEDs, a great deal of efficient device structures have been reported. One of the most successful strategies is the adoption of a carrier- and exciton-confining structure in WOLEDs by Su et al., in which the device showed high efficiency and reduced efficiency roll-off [133]. The WOLED had the configuration of ITO/MCC-PC (20 nm)/bis(m-di-p-tolylaminophenyl)-1,10-biphenyl (3DTAPBP, 20 nm)/TCTA: FIrpic (4.75 nm)/TCTA: PQIr (0.25 nm)/DCzPPy: PQIr (0.25 nm)/DCzPPy: FIrpic (4.75 nm)/1,3-bis(3,5-dipyrid-3-yl-phenyl)benzene (BmPyPB, 50 nm)/LiF/Al, as shown in Figure 10a. Without the use of any outcoupling techniques, the device showed a PE of 53 lm W^−1^ at 100 cd m^−2^ in the forward direction, and rolled off slightly to 44 lm W^−1^ at 1000 cd m^−2^, which were the highest values ever observed for WOLEDs at that time. Factors contributing to the efficient white-light emission are: (1) To obtain low operating voltage, thin EMLs (10 nm) and a stepped progression of the HOMO and LUMO energy levels of the HTL (3DTAPBP), EML [TCTA: FIrpic (5 nm)/DCzPPy: FIrpic (5 nm)], and ETL (BmPyPB) were employed, ensuring a high PE; (2) A double-EML and a bipolar host DCzPPy were employed to broaden the exciton-formation zone, and thus reduce efficiency roll-off; (3) The HTL (3DTAPBP) had a wide energy gap of 3.57 eV and high-lying LUMO of 2.13 eV, while the ETL (BmPyPB) possessed a wide energy gap of 4.05 eV and low-lying HOMO of 6.67 eV, which could effectively confine carriers and triplet excitons, as shown in Figure 10b. By optimizing the device structure, a FIrpic-based blue OLED exhibited a PE of 46 lm W^−1^ at 1000 cd m^−2^, which was the highest PE achieved for a blue electrophosphorescent device at that time. After obtaining the efficient blue device, they inserted two ultrathin orange EMLs TCTA: PQIr (0.25 nm)/DCzPPy: PQIr (0.25 nm) between the double EMLs of the blue OLED, generating the white emission. Moreover, the CIE coordinates were (0.341, 0.396) at 100 cd m^−2^, with a shift slightly to (0.335, 0.396) at 1000 cd m^−2^, indicating a stable color.

#### 4.2.2. Combining Blue EML Surrounded by Green/Red EML Structure and Outcoupling Technology

In general, the exciton energy levels (T_1_ and S_1_) of blue phosphors are higher than those of green or red phosphors. Hence, the energy on blue phosphors can be transferred to low-energy green or red phosphors. In this way, the blue host-guest system is positioned at the main exciton formation region and then surrounded by green/red EMLs, which can completely harvest the generated excitons.

**Figure 11:**
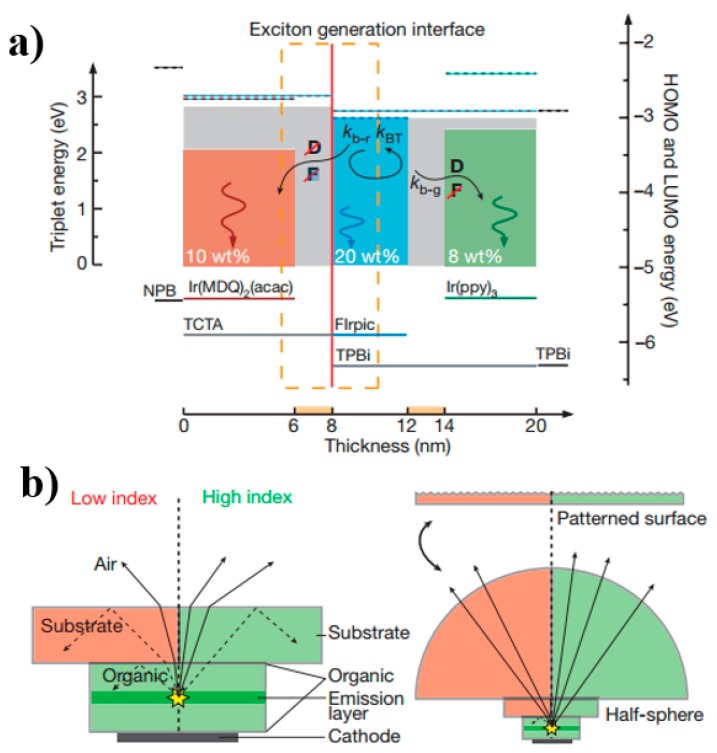
(**a**) Device structure of the WOLED and the proposed energy level. (**b**) Left: a cross-section of an OLED to illustrate the light propagation. Solid lines indicate modes escaping the device to the forward hemisphere; dashed lines represent trapped modes. Right: a large half-sphere and a patterned surface can be applied to increase light outcoupling. Reproduced from Reference [134].

In 2009, Reineke et al. combined this rational strategy and outcoupling technology, demonstrating a WOLED with fluorescent tube efficiency for the first time [134]. Figure 11a depicts the structure: ITO/MeO-TPD: NDP-2 (60 nm)/NPB (10 nm)/TCTA: 10% Ir(MDQ)_2_(acac) (6 nm)/TCTA (2 nm)/TPBi: 20% FIrpic (4 nm)/TPBi (2 nm)/TPBi: 8% Ir(ppy)_3_ (6 nm)/TPBi (10 nm)/Bphen: Cs/Al, where Bphen is 4,7-diphenyl-1,10-phenanthroline, and Ir(MDQ)_2_(acac) is iridium(III) bis(2-methyldibenzo[f,h]quinoxaline) (acetylacetonate). The high performance could be attributed to the following factors: (1) The blue phosphor was positioned within the EML and its combination with a carefully chosen host material, leading to the exciton formation region being at the interface of a double-EML structure; (2) The blue host-guest system was surrounded by red and green sublayers of the EML to harvest unused excitons; (3) Holes and electrons were injected without facing any energy barrier into the EML from NPB to TCTA: Ir(MDQ)_2_(acac) and from TPBi to TPBi: Ir(ppy)_3_, respectively, which kept the operating voltage low; (4) The outermost layers in contact with the electrodes were chemically p- and n-doped, which reduced ohmic losses to a negligible level; (5) 2 nm TCTA and 2 nm TPBi were used as the thin intrinsic interlayers to separate the different sublayers, decoupling the sublayers from unwanted Förster energy transfer. Therefore, excitons created in the blue region on host or dopant had various decay channels. Without outcoupling enhancement, 30 and 33 lm W^−1^ were achieved in a forward direction of 1000 cd m^−2^, respectively, which corresponds to 13.1% EQE for a device with low-refractive-index glass substrates (CRI = 80, CIE: (0.45, 0.47)) and 14.4% EQE for a device with high-refractive-index substrates (CRI = 80, CIE: (0.44, 0.46)).

For the different efficiency, it can be attributed to the fact that high-refractive-index substrates can substantially increase the amount of light coupled from the organic layers into the glass substrate (up to 80%), as shown in Figure 11b left. This is because light will partly be reflected because of total internal reflection at the organic (refractive index *n*: 1.7–1.9)/substrate (*n*: 1.51) interface (i.e., organic modes) and substrate/air (*n*: 1) interface (i.e., substrate modes), if a low-refractive-index substrate is used. By increasing *n* of the substrate to 1.78, the index mismatch between organic materials and substrate vanishes, enhancing light coupling into the high-refractive-index glass. Therefore, all photons guided to organic modes by total internal reflection at the organic/glass interface in the low-refractive-index case are entering the glass substrate. To further enhance the efficiency, shaped substrates have been used (i.e., a pattern of pyramids with period of 0.5 mm achieved by cutting 90° grooves into a high index glass, Figure 11b right), which enables the coupling of light under high angles of incidence (to the substrate surface normal). As a consequence, device with high-refractive-index substrates achieves 26% EQE and 63 lm W^−1^. Moreover, to avoid plasmonic losses to the metal (i.e., the emitting dipoles couple to surface plasmons of the reflective metal), the efficiency can be increased further by placing the EML further away from the reflective cathode. This is because plasmonic losses are the dominating loss channel when the emission takes place in the proximity of the metal. This impact decreases with greater distances between the EML and cathode, and drops to a negligible level for distances >200 nm. Besides, if the EML is placed in the second antinode of the reflective metal cathode, OLEDs exhibit a more direct emission, which makes the light outcoupling of substrate modes easier. Hence, 205 and 200 nm ETLs have been prepared to best fit the second outcoupling maximum. Taking the three outcoupling strategies into account (i.e., reducing the organic modes, substrate modes, and plasmonic losses), 90 lm W^−1^ (34% EQE, CRI = 69, CIE: (0.41, 0.49)) and 87 lm W^−1^ (34% EQE, CRI = 72, CIE: (0.43, 0.49)) are achieved at 1000 cd m^−2^ for a device with high-refractive-index substrates comprising 205 and 210 nm ETLs, respectively. Therefore, compared with the WOLED without outcoupling technology, the efficiency of the WOLED with outcoupling technologies shows ~300% enhancement. In a word, by using the blue EML surrounded by a green/red EML structure and outcoupling technology, for the first time, WOLEDs with efficiencies approaching 100 lm W^−1^ even at high brightness are possible.

#### 4.2.3. Single-Host System Structure

Since the synthesis of universal hosts is still a challenge, several different hosts are usually needed for multi-EML phosphorescent WOLEDs (e.g., three hosts for blue, green, and red guests). As a consequence, more structural heterogeneity, more evaporation sources, longer fabrication processes, and more device engineering methods are necessary. Therefore, the introduction of a single host is an alternative strategy that can alleviate these difficulties.

**Figure 12:**
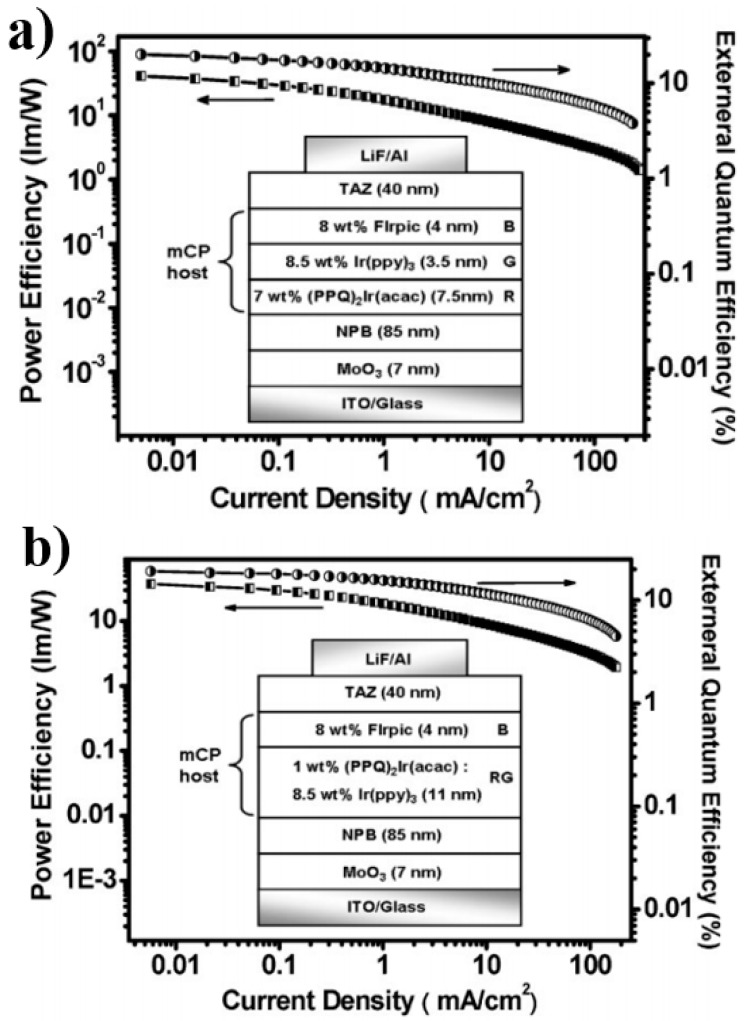
The WOLED architecture/efficiency of (**a**) the first device; (**b**) the second device. Reproduced from Reference [135].

Wang et al. used mCP as the single host, arranged the place of three primary-color emitters and manipulated the charges and excitons distribution, developing two high-efficiency, high-CRI, and stable-color WOLEDs [135]. As displayed in Figure 12, the first WOLED has a “blue-green-red” structure: ITO/MoO_3_/NPB/mCP: 7% bis(2,4-diphenylquinolyl-*N*,*C*^2^’)iridium(acetylacetonate) [(PPQ)_2_Ir(acac), 7.5 nm)]/mCP: 8.5% Ir(ppy)_3_ (8.5 nm)/mCP: 8% FIrpic (4 nm)/TAZ/LiF/Al, while the second WOLED has a “blue-green/red” structure: ITO/MoO_3_/NPB/mCP: 8.5% Ir(ppy)_3_: 1% (PPQ)_2_Ir(acac) (11 nm)/mCP: 8% FIrpic (4 nm)/TAZ/LiF/Al. The first white device exhibited a maximum forward-viewing PE of 41.3 lm W^−1^, EQE of 20.1%, CRI of 85, and small CIE variation of (0.01, 0.01) during 500–10,000 cd m^−2^. The second white device showed a maximum forward-viewing PE of 37.3 lm W^−1^, EQE of 19.1%, CRI of 80, and no color shift of (0.00, 0.00) during 500–10,000 cd m^−2^. The reasons for the high performance can be explained as follows: (1) The distribution of different emitters in multiple regions within a single-host system reduces structural heterogeneity and facilitates charge injection and transport between different emissive centers. Moreover, it allows flexible manipulation of each emissive centers as well as precious control of the exciton or charge to enhance the EL performance; (2) Placing the high-energy blue phosphor closest to the main recombination zone, followed by the long and longer wavelength phosphors, ensures that the excitons formed can diffuse throughout the emissive region to produce a desired output color balance. Additionally, the exciton-formation region can be broadened, and thus enhance the device efficiency by lowering the local triplet accumulation; (3) For the first WOLED, due to the high concentration of red phosphor (7%), (PPQ)_2_Ir(acac) could easily trap the charges. Hence, with the voltage increasing, (PPQ)_2_Ir(acac) could harvest more excitons to generate a red emission, leading to the red-shifted color. To stabilize the color, the low concentration (1%) (PPQ)_2_Ir(acac) was doped into the green EML in the second WOLED, forming a mixed green/red WML. As a result, the red emission generated by the self-charge-trapping effect of (PPQ)_2_Ir(acac), obtaining stable colors.

#### 4.2.4. Triplet Exciton Conversion Structure

**Figure 13:**
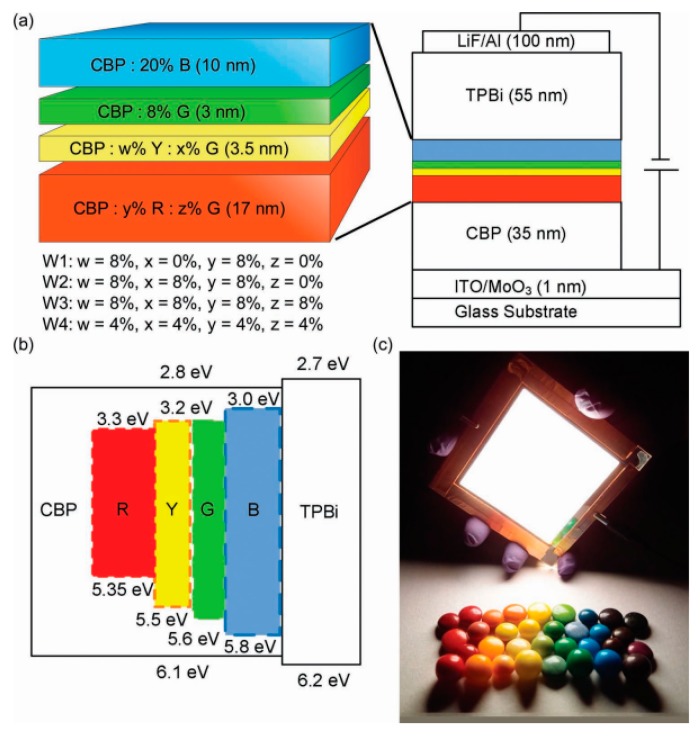
(**a**) The device structure; (**b**) The energy level diagrams; (**c**) A photo of a large area (80 mm × 80 mm) WOLED illuminating at 5000 cd m^−2^ with a CRI of 85. Reproduced from Reference [136].

For multi-EML phosphorescent WOLEDs, the careful management of the energy transfer between the adjacent EMLs is necessary for achieving high performance, since the high-energy excitons on phosphors with shorter wavelengths are readily transferred to low-energy phosphors with longer wavelengths. In 2003, Chang et al. proposed a triplet exciton conversion strategy to develop four-color WOLEDs, in which the emission efficiency of yellow and red phosphors is determined by the green phosphor [136]. In their device, the key for the high efficiency was the effective exciton gathering by the green phosphor followed by efficient energy transfer to an emitter with a lower energy. Figure 13 depicts the WOLED architecture: ITO/MoO_3_ (1 nm)/CBP: Ir(MDQ)_2_(acac): iridium(III) bis(2-phenylpyridine)-(acetylacetonate) [Ir(ppy)_2_(acac), 17 nm]/CBP: iridium(III) bis(2 phenylbenzothiozolato *N*,*C*^2^′)(acetylacetonate) [Ir(BT)_2_(acac)]: Ir(ppy)_2_(acac) (3.5 nm)/CBP: Ir(ppy)_2_(acac) (3 nm)/CBP: FIrpic (20 nm)/TPBi (55 nm)/LiF/Al. The device exhibited an EQE of 24.5% at 1000 cd m^−2^ with a CRI of 81, and an EQE of 20.4% at 5000 cd m^−2^ with a CRI of 85. The high performance can be explained as follows: (1) The main exciton generation zone was located near the CBP/TPBi interface; in addition, both CBP and TPBi possess a wide energy gap as well as high T_1_, confining excitons within emitters; (2) Since FIrpic has the closest energy levels to CBP and TPBi, direct exciton formation on FIrpic is unlikely, and it is critical to place FIrpic closest to the CBP/TPBi interface to harvest excitons first; (3) Lower-energy green, yellow, and red emitters were placed sequentially next to FIrpic to harvest excitons in a cascaded fashion in the single CBP host. Hence, only a single site for exciton generation and recombination was available, as no other barrier layers that could induce charge accumulation were introduced, avoiding TPQ and polaron-polaron quenching; (4) Since there was no interlayer between adjacent EMLs, surplus excitons readily diffused into the adjacent layer with a lower energy emitter, maximizing the efficiency.

#### 4.2.5. Incorporating Outcoupling Technology with Energy Cascade Structure 

Since there is a limit to improving the material efficiency and fabrication technology, the incorporation of outcoupling technology with an efficient structure is an effective strategy. Therefore, it is very important to improve the light extraction efficiency by improving outcoupling from the conventional device, because the light outcoupling is only ~20% in the conventional OLED architecture [137,138,139,140]. In general, the majority of internally generated light is confined in the substrate and waveguide modes due to the index mismatch of multiple layers, or is lost by absorption and surface plasmon polaritons of metal electrodes [141,142,143,144,145,146].

**Figure 14:**
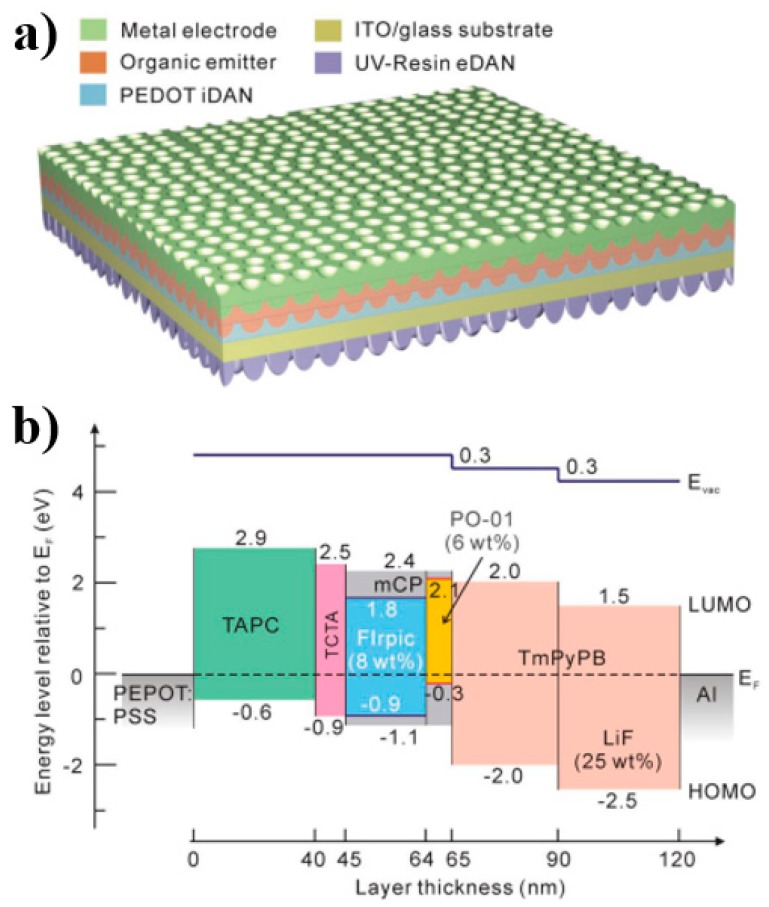
(**a**) Schematic of the WOLED; (**b**) Energy level diagram of the organic emitter with the alignment of the Fermi level across the interface. Reproduced from Reference [9].

Ou et al. used a novel approach for broadband quasi-omnidirectional light extraction with reduced energy loss during electron-photon conversion [9]. As shown in Figure 14a, both sides of the ITO glass substrate were patterned with deterministic aperiodic nanostructures (DANs). Then, organic layers and the metal electrode were deposited onto the substrate. Therefore, the key feature is using DANs with long-range disorder and short-range order that significantly reduce the optical confinement without spectral distortion. When utilizing the nanostructured ITO glass with DANs, a higher value on transmittance with ~7% enhancement was yielded over the entire visible spectrum. This is because the broad distribution of periodicity and the gradient refractive index profiles of the sub-wavelength DAN pattern can fully interact with incident light and enable the reduced reflection loss. For the function of DANs, the internal DAN promotes the outcoupling of the waveguide-mode light from the organic layers and ITO electrode into the glass substrate, while the external DAN enables the extraction of light trapped in glass due to the total internal reflection. As a result, this outcoupling technology can greatly enhance the efficiency (2.31 times).

Further, they proposed a multilayer energy cascade structure of ITO/PEDOT: PSS/TAPC (40 nm)/TCTA(5 nm)/FIrpic: mCP (8 wt %, 19 nm)/PO-01: mCP (6 wt %, 1 nm)/TmPyPB (25 nm)/LiF: TmPyPB (25 wt %, 30 nm)/LiF (0.7 nm)/Al (100 nm), as shown in Figure 14b. For this structure, the insertion of an ultrathin TCTA and an n-doped EIL (LiF: TmPyPB) was crucial to the device performance. Moreover, the multilayer emitter was nearly barrier-free with the reduction of ohmic losses on a negligible level, which can energetically facilitate hole transport from TAPC to FIrpic: mCP and electron transport through TmPyPB to PO-01: mCP, respectively. Without the outcoupling enhancement, the WOLED exhibited the EQE and PE of 23.6% and 60.0 lm W^−1^ at 1000 cd m^−2^ (<4 V), respectively. By incorporating the novel outcoupling technology with the efficient structure, the WOLED exhibited the EQE and PE of 54.6% and 123.4 lm W^−1^ at 1000 cd m^−2^, respectively, which represents the most efficient WOLED in the revealed literature so far. Moreover, this OLED structure offers an extremely small efficiency roll-off at high luminance and superior angular color stability over the visible wavelength range.

## 5. TADF WOLEDs

### 5.1. WOLEDs with All TADF Emitters

Similar to phosphorescent WOLEDs, TADF WOLEDs show promise in achieving high performance, since TADF emitters can (i) harness triplet excitons; (ii) exhibit excellent efficiency; (iii) have intrinsically advantages in obtaining high T_1_ because of the decreased singlet-triplet splits; (iv) usually show broad emission spectra with a rather large full width at half-maximum of about 100 nm, which is wider than that of conventional fluorescent materials because of their charge-transfer nature [147,148,149,150,151,152,153,154,155].

**Figure 15:**
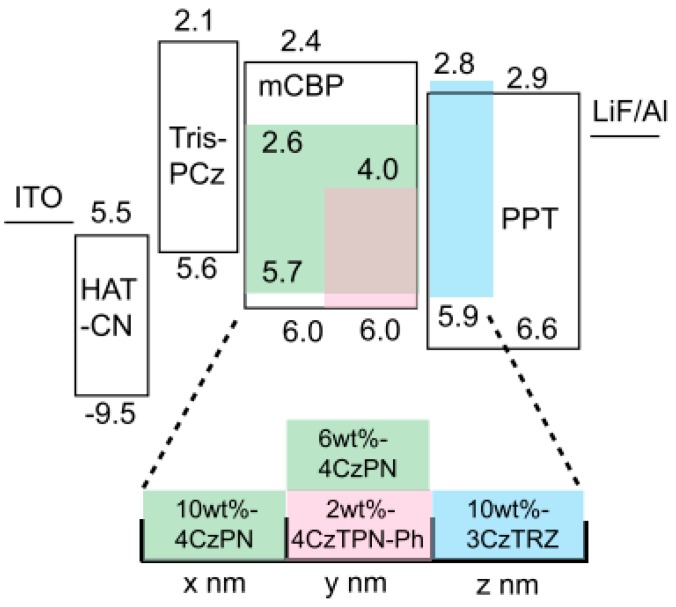
The WOLED structures and energy level diagram. Reproduced from Reference [156].

To develop TADF WOLEDs, a rational strategy is the combination of blue, green, and red TADF emitters. In 2014, Adachi et al. used this strategy to realize a high-efficiency TADF WOLED [156]. Figure 15 depicts the device structure: ITO/1,4,5,8,9,11-hexaazatriphenylene hexacarbonitrile (HAT-CN, 10 nm)/9,9′,9′′-triphenyl-9*H*,9′*H*,9′′*H*-3,3′:6′,3′′-tercarbazole (Tris-PCz, 35 nm)/10 wt % 1,2,3,4-tetrakis(carbazol-9-yl)-5,6-dicyanobenzene (4CzPN): 3,3-Di(9*H*-carbazol-9-yl)biphenyl (mCBP, green EML) (*x* nm)/6 wt % 4CzPN: 2 wt % 41,4-dicyano-2,3,5,6-tetrakis(3,6-diphenylcarbazol-9-yl)benzene (CzTPN-Ph): mCBP (red EML) (*y* nm)/10 wt % 9-(3-(9*H*-carbazol-9-yl)-9-(4-(4,6-diphenyl-1,3,5-triazin-2-yl)phenyl)-9*H*-carbazol-6-yl) -9*H*-carbazole (3CzTRZ): 2,8-bis(diphenylphosphoryl) dibenzo-[b,d] thiophene (PPT, blue EML) (*z* nm)/PPT (50 nm)/LiF/Al. By optimizing the charge generation zone via the adjustment of different EML thicknesses (the total thickness was set to be *x* + *y* + *z* = 15 nm), the WOLED achieved a maximum EQE of over 17% with CIE coordinates of (0.30, 0.38).

### 5.2. WOLEDs with TADF and Conventional Fluorescent Emitters

Given that TADF can harvest triplet excitons, the combination of TADF and conventional fluorescent emitters in WOLEDs has potential to achieve 100% IQE. In this strategy, a TADF molecule acts as a triplet harvester for other-color fluorescent emitters to achieve white emission [157,158,159].

**Figure 16:**
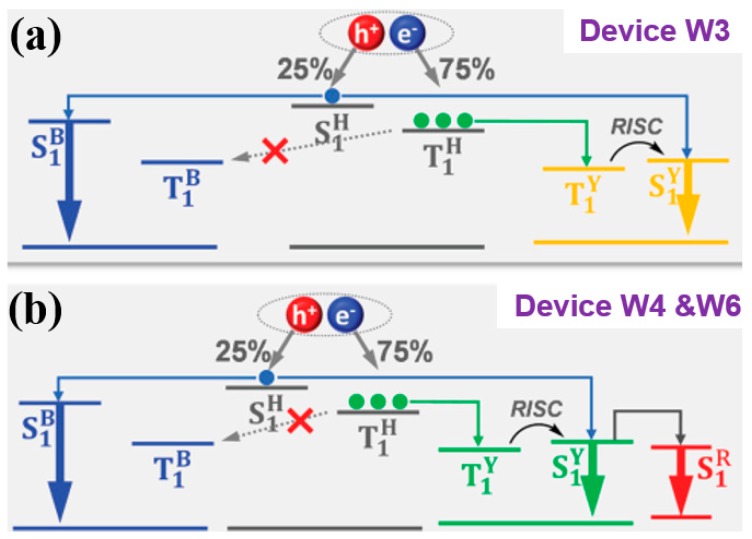
Device function mechanisms of the conceptual utilization of singlet and triplet excitons generated in EMLs. (**a**) Device W3, and (**b**) devices W4 and W6. H, B, Y, and R denote CBP, NI-1-PhTPA, PXZDSO2 and DBP, respectively. Reproduced from Reference [160].

For example, Li et al. reported high-efficiency and high CRI WOLEDs with the chromaticity-adjustable yellow TADF emitter 2-(4-phenoxazinephenyl)thianthrene-9,9′,10,10′-tetraoxide (PXZDSO2) [160]. By combining the conventional deep-blue-fluorescence emitter NI-1-PhTPA and PXZDSO2, the two-color WOLED showed a maximum EQE of 15.8% (device W3). Then, since the chromaticity of the EML containing PXZDSO2 could be tuned to yellowish green, they introduced a deep-red-fluorescence emitter DBP (dibenzo{[f,f′]-4,4′,7,7′-tetraphenyl}diindeno[1,2,3-cd:1′,2′,3′-lm]perylene) to fabricate a three-color WOLED, achieving the most efficient ever EQE of 19.2% with a CRI of 68 (device W4), and the highest ever CRI of 95 with an EQE of 15.6% (device W6). The configurations are ITO/HATCN/TAPC/EMLs/TmPyPB/LiF/Al, in which device W3 has an EML of CBP: 8 wt % NI-1-PhTPA (10 nm)/CBP (3 nm)/CBP: 6 wt % PXZDSO2 (15 nm)/CBP (3 nm)/CBP: 8 wt % NI-1-PhTPA (10 nm), device W4 has an EML of CBP: 7 wt % NI-1-PhTPA (10 nm)/CBP (3 nm)/CBP: 3 wt % PXZDSO2 (5 nm)/CBP: 5 wt % PXZDSO2: 0.3 wt % DBP (5 nm)/CBP: 3 wt % PXZDSO2 (5 nm)/CBP (3 nm)/CBP: 7 wt % NI-1-PhTPA (10 nm), and device W6 has an EML of CBP: 10 wt % NI-1-PhTPA (10 nm)/CBP (3 nm)/CBP: 5 wt %PXZDSO2: 0.35 wt % DBP (15 nm)/CBP (3 nm)/CBP: 10 wt % NI-1-PhTPA (10 nm). The device working mechanisms are described as follows. For device W3, (1) since NI-1-PhTPA is a deep-blue-fluorescence emitter and CBP: 6 wt % PXZDSO2 emit yellow light with a broad spectrum, high-performance two-color WOLEDs were realized; (2) given the almost equal T_1_ level of NI-1-PhTPA and PXZDSO2, efficiency roll-off occurred if they were in direct contact due to triplet exciton quenching by NI-1-PhTPA; (3) the efficiency roll-off could be further induced as the formed triplet excitons of NI-1-PhTPA cannot be utilized by PXZDSO2; (4) to stabilize the recombination zone that occurs in whole EMLs since NI-1-PhTPA/CBP are bipolar and to avoid triplet exciton quenching by NI-1-PhTPA, two 3-nm CBPs were inserted between the blue and yellow EMLs, restraining the inevitable Förster energy transfer from NI-1-PhTPA to PXZDSO2; (5) to reduce the triplet exciton energy loss via a nonradiative transition process, blue-fluorescence emitter was dispersed in CBP for blue emission, leading to most excitons being generated at CBP; (6) triplet energy transferred from CBP gives most triplet excitons of PXZDSO2 since triplet excitons typically have long diffusion lengths (≈100 nm), as shown in Figure 16a. Hence, an EQE of 15.8% was achieved for device W3. For device W4, (1) a deep-red-fluorescence emitter DBP was conceived to be used; (2) PXZDSO2 was an assistant host for DBP to realize red light emission due to the efficient energy transfer from the S_1_ of PXZDSO2; (3) the doping concentration of PXZDSO2 was decreased to reduce intermolecular aggregation and thus blue shifted emission (20 nm), achieving green emission, complementary to emissions of NI-1-PhTPA and DBP; (4) a red EML of CBP: 5 wt % PXZDSO2: 0.3 wt % DBP was inserted between the two green EMLs of CBP: 3 wt % PXZDSO2 to receive singlet exciton energy transferred from the PXZDSO2 molecules in both sides to give both green and red emissions; (5) the two doped blue EMLs and CBP interlayers were located at the both sides of the green EMLs to give blue emission and to confine the PXZDSO2 triplet excitons, respectively, as shown in Figure 16b. Thus, an EQE of 19.2% was achieved for device W4. Furthermore, an EML consisting of improved DBP doping concentration was utilized instead of the green and red EMLs for candle-style warm WOLEDs (device W6), achieving a high CRI of 95.

## 6. Hybrid WOLEDs

### 6.1. Types of Hybrid WOLEDs

By combining blue fluorophores and yellow/orange or green-red phosphors, hybrid WOLEDs can be developed. In 2003, Li et al. reported the first hybrid WOLED [161]. Since then, more and more attention has been paid to this type of WOLED due to its utilization of blue fluorophores and high exciton-harvesting efficiency. As a matter of fact, most available products in the WOLED market have now adopted the hybrid WOLED technology.

**Figure 17:**
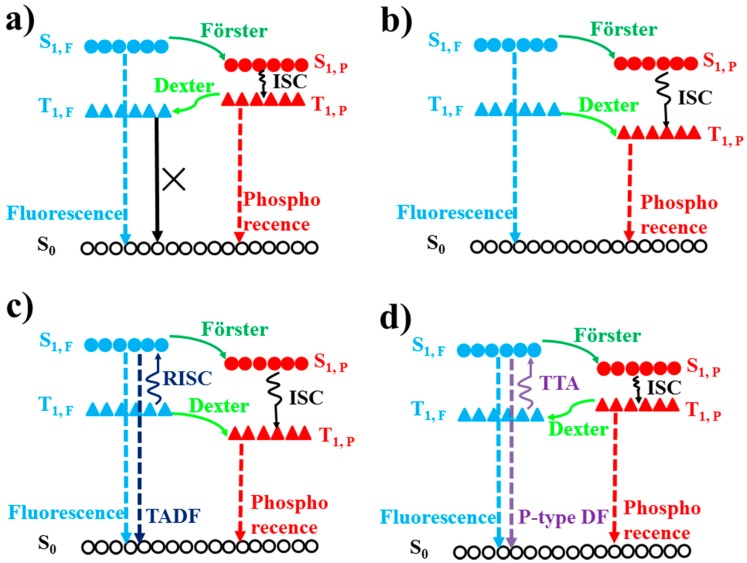
Emission mechanisms for different hybrid WOLEDs and the possible methods of decay of singlet and triplet excitons. (**a**) Hybrid WOLEDs with low-T_1_ blue fluorophores; (**b**) Hybrid WOLEDs with high-T_1_ blue fluorophores; (**c**) Hybrid WOLEDs with TADF blue fluorophores; (**d**) Hybrid WOLEDs with p-type blue fluorophores. S_1,F_ and T_1,F_ represent the singlet and triplet energy of fluorophores, respectively. S_1,P_ and T_1,P_ represent the singlet and triplet energy of phosphors, respectively. S_0_ represents the ground state. DF is delayed fluorescence. Förster and Dexter represent the Förster and Dexter energy transfer between fluorophores and phosphors, respectively.

For hybrid WOLEDs, realizing 100% exciton-harvesting efficiency is the main focus. On one hand, for hybrid WOLEDs with conventional blue fluorophores, singlets should be harvested by fluorescent emitters while triplets should be harvested by phosphorescent emitters, as shown in Figure 17a,b. On the other hand, for hybrid WOLEDs with TADF or p-type blue fluorophores, since blue fluorophores can harvest both singlet and triplet excitons, excitons can be readily harvested by either blue fluorophores or the complementary phosphors, as shown in Figure 15c,d.

The T_1_ of fluorescent emitters has a significant influence on hybrid WOLEDs, because it will affect the design of device structures and the selection of materials, particularly for hybrid WOLEDs with conventional blue fluorophores [162,163,164,165,166,167,168,169,170,171]. Simply, based on the T_1_ of blue fluorophores, two types of hybrid WOLEDs are classified. For the first one, the T_1_ of blue fluorescent emitters is below the T_1_ of phosphors (Figure 17a). By inserting interlayers (also known as spacers) between the fluorescent and phosphorescent EMLs, (1) the Förster energy transfer from the fluorescent EMLs to phosphorescent EMLs is prevented; (2) Dexter energy transfer from phosphorescent EMLs to fluorescent EMLs is eliminated, thus preventing triplet excitons from being quenched by fluorophores; (3) emission colors can be tuned (e.g., adjusting the thickness of interlayers, changing the cooping ratio of mixed interlayers); and (4) the lifetime can be prolonged [172,173,174]. For the latter one, the T_1_ of blue fluorescent emitters is above the T_1_ of phosphors (Figure 17b) [175,176,177,178]. In this case, blue fluorescent EMLs are generally located near the exciton generation zone. As a result, singlet excitons are harnessed via fluorescent EMLs for blue light. For these unutilized triplet excitons, they will be harnessed by nearby phosphors via the diffusion mechanism, because triplet excitons possess a much longer lifetime as well as a longer diffusion distance compared with singlet excitons. Thus, despite having no interlayers, 100% exciton-harvesting efficiency can be realized via the manipulation of charges and excitons distribution, leading to the unity IQE. In the case of hybrid WOLEDs with TADF blue fluorophores (Figure 17c), triplet excitons formed onto blue TADF fluorophores can be readily harvested either by phosphors via the Dexter energy transfer due to the high T_1_ of blue TADF fluorophores, or by the emitting singlet state via the RISC process. For hybrid WOLEDs with p-type blue fluorophores (Figure 17d), triplet excitons formed onto p-type blue fluorophores are harnessed by the emitting singlet state via the TTA procedure. Moreover, even regarding triplet excitons on phosphors trapped by p-type blue fluorophores with a low T_1_, these triplet excitons can be recycled via the TTA procedure.

### 6.2. Low-T_1_ Fluorophore-Based Hybrid WOLEDs

#### 6.2.1. Bipolar Interlayer-Based Hybrid WOLEDs

In the case of low-T_1_ fluorophore-based hybrid WOLEDs, introducing bipolar interlayers between fluorescent and phosphorescent EMLs is an efficient strategy to prevent mutual exciton quenching, ensuring high performance. For bipolar materials that can function as the interlayers, their hole and electron mobility should be almost the same. Besides, the T_1_ of bipolar materials should be high enough, otherwise excitons could be quenched by the interlayers.

**Figure 18:**
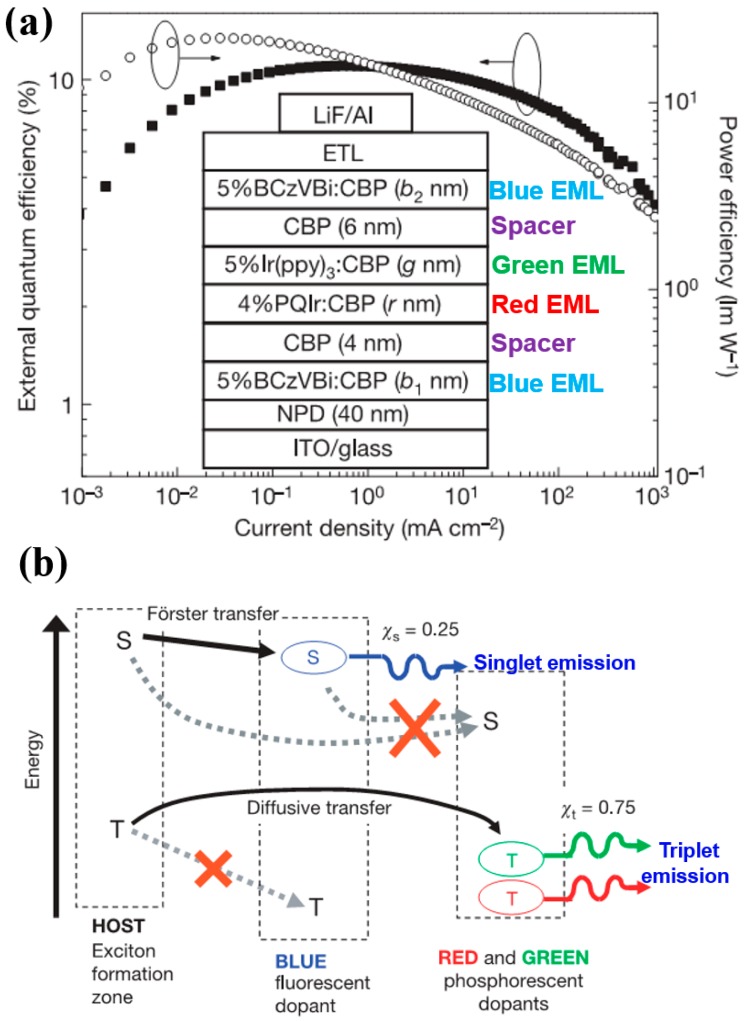
(**a**) The WOLED architecture and efficiency. (**b**) Diagrams of EL procedures. Reproduced from Reference [184].

To date, CBP is the most popular bipolar interlayer, because (1) its electron mobility (10^−4^ cm^2^ V^−1^ s^−1^) is almost similar to its hole mobility (10^−3^ cm^2^ V^−1^ s^−1^); and (2) its T_1_ is as high as 2.56 eV, which is high enough for green phosphorescent emitters (~2.4 eV), prohibiting exciton quenching [179,180,181,182,183]. In fact, the first groundbreaking hybrid WOLED with high performance was designed via the use of a CBP interlayer [184]. Figure 18a depicts the WOLED structure: ITO/NPD (40 nm)/CBP: 5% 4,4′-bis(9-ethyl-3-carbazovinylene)-1,1’-biphenyl (BCzVBi)/CBP (4 nm)/CBP: 4% iridium(III) bis(2-phenyl quinolyl-*N*,*C*^2^’)acetylacetonate (PQIr)/CBP: 5% Ir(ppy)_3_/CBP (6 nm)/CBP: 5% BCzVBi/ETLs/LiF/Al. The WOLED function mechanism can be summarized as follows: (1) Excitons were firstly generated onto a CBP host, then singlet excitons were transfered to BCzVBi via the Förster procedure; (2) For unused triplet excitons on host CBP, they would diffuse to phosphorescent EMLs because of the ~100 nm diffusion lengths and then transfer to phosphorescent emitters Ir(ppy)_3_ and PQIr; (3) As displayed in Figure 18b, the energy could not be directly transferred from BCzVBi to Ir(ppy)_3_ or PQIr, because the interlayer CBP was set to be thicker than the Förster radius of ~3 nm. Thus, singlets and triplets could be harnessed via totally independent channels, in which singlet excitons could be harnessed by BCzVBi for blue emission while triplet excitons could be harnessed by Ir(ppy)_3_ and PQIr for green and red light, respectively. As a result, the WOLED exhibited a total EQE and PE of 18.7% and 37.6 lm W^−1^, respectively.

**Figure 19:**
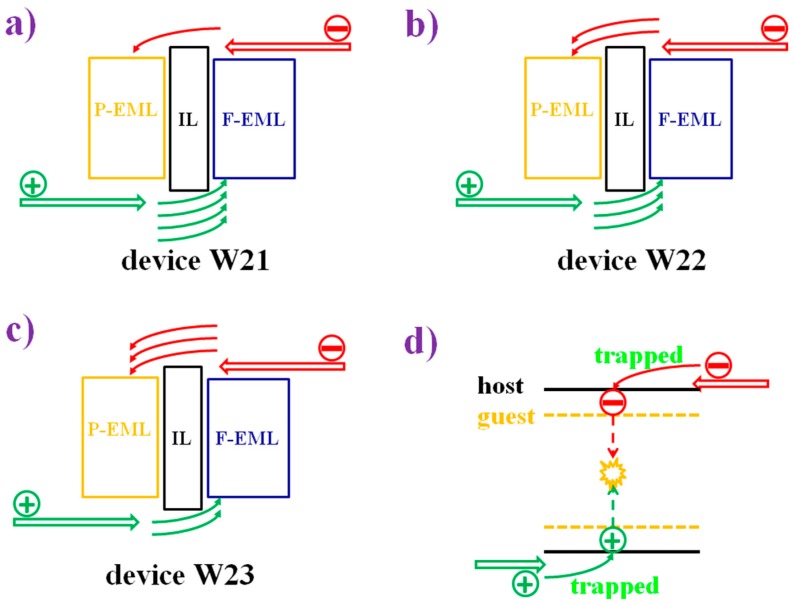
The WOLEDs’ function mechanism. IL is interlayer. F-EML is fluorescent EML. P-EML is phosphorescent EML. (**a**) TAPC:TmPyPB = 1:0; (**b**) TAPC:TmPyPB = 0.8:0.2; (**c**) TAPC:TmPyPB = 0.5:0.5; (**d**) Charge trapping effect of Ir(dmppy)_2_(dpp). Reproduced from Reference [193].

The use of co-doped bipolar interlayers is also an effective strategy to manipulate the charge and exciton distribution, guaranteeing high performance. By co-doping p-type materials with n-type materials at an appropriate ratio, the performance of a hybrid WOLED can be optimized. In addition, considering that lots of high-T_1_ p-type or n-type materials have been reported, the use of co-doped bipolar interlayers is very flexible [185,186,187,188,189,190,191,192]. For instance, by co-doping the p-type material TAPC with the n-type material TmPyPB, a co-doped bipolar interlayer was used to organize the high-performance hybrid WOLEDs by Liu et al. [193]. The device structure of two-color WOLEDs is as follows: ITO/HAT-CN (100 nm)/NPB (15 nm)/TAPC (5 nm)/NPB: Bepp_2_: Ir(dmppy)_2_(dpp) (25 nm)/interlayer (4 nm)/9,10-Bis[4-(1,2,2-triphenylvinyl)phenyl]anthracene (BTPEAn, blue aggregation-induced emission (AIE) fluorophores, 10 nm)/Bepp_2_ (40 nm)/LiF/Al, in which different co-doping ratios of TAPC: TmPyPB interlayer were used. Two-color devices can show (1) highly efficient and stable pure-white color (32.0 lm W^−1^); (2) 38.9 lm W^−1^ as well as reduced efficiency roll-off; or (3) a very high efficiency of 70.2 cd A^−1^ and 43.4 lm W^−1^ at a very high luminance of 10,000 cd m^−2^. The influence of the co-doped bipolar interlayer TAPC: TmPyPB was studied to unveil the factors necessary for excellent performance. As shown in Figure 19a, in the case of device W21 with TAPC:TmPyPB = 1:0, holes are readily transported to fluorescent EML due to the p-type property of TAPC. However, relatively few electrons are transported to phosphorescent EML by virtue of the tunneling effect. As a result, the pure-white color is obtained because of the same yellow and blue intensity. As shown in Figure 19b, in the case of W22 with TAPC:TmPyPB = 0.8:0.2, electrons are more easily transported to phosphorescent EML, leading to the stronger yellow intensity. As shown in Figure 19c, for W23 with TAPC:TmPyPB = 0.5:0.5, it becomes difficult for holes to cross this layer, resulting in excitons being easily recombined in the phosphorescent region. As a result, white emissions can be produced only when the voltage is high enough to provide the high energy. The reason for the stronger yellow emission can be further explained as follows: (1) The fluorescent EML is set to be thinner than the phosphorescent one; and (2) Charges are readily trapped by Ir(dmppy)_2_(dpp) due to this material’s higher HOMO and lower LUMO compared with those of NPB and Bepp_2_ hosts, as displayed in Figure 19d. Furthermore, the sunlight-like color is realized by the three-color device possessing a CCT span of 2328–10,690 K.

#### 6.2.2. Unipolar Interlayer-Based Hybrid WOLEDs

Based on different device architectures, interlayers should be managed accordingly in order to yield high performance. Hence, unipolar materials (n-type and p-type) can be more efficient for manipulating the charges and excitons distribution in some structures compared with their bipolar counterparts, if the EMLs possess unipolar transporting properties [194].

**Figure 20:**
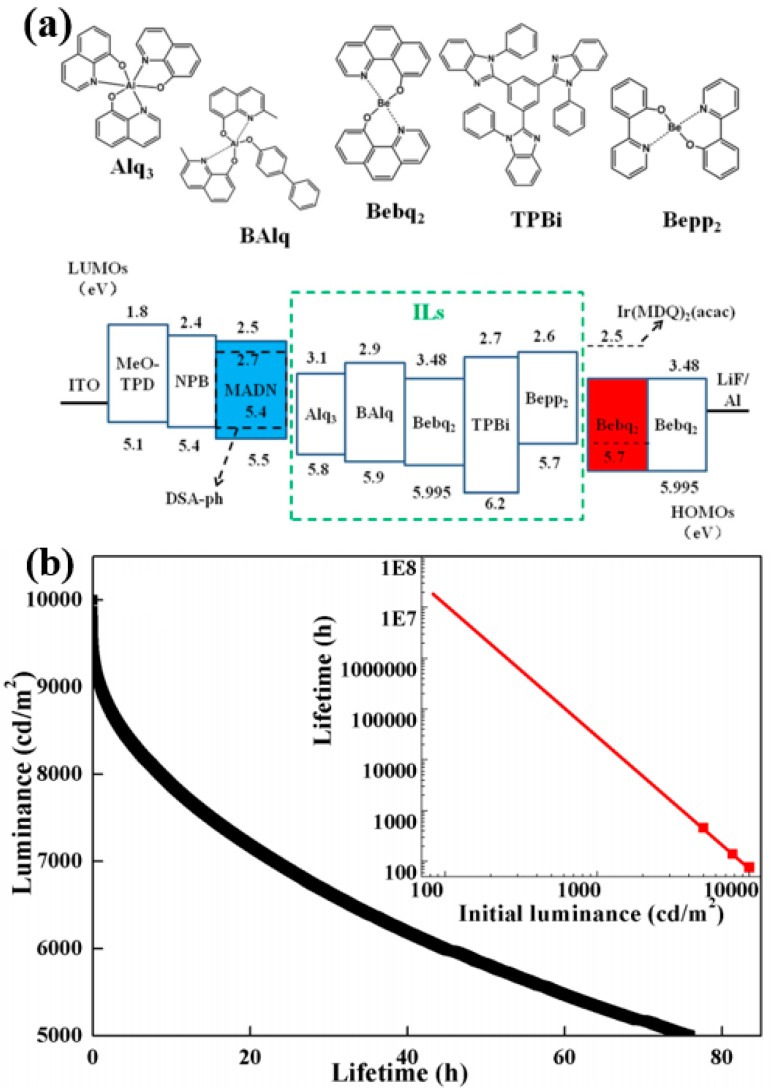
(**a**) n-type materials chemical structures, the WOLED structure and diagram of energy levels; (**b**) The WOLED lifetime. Reproduced from Reference [198].

The introduction of n-type interlayers was demonstrated to be an effective strategy to develop high-performance hybrid WOLEDs [195,196,197]. For example, the influence of n-type interlayers was investigated by Liu et al. [198]. In their devices, the n-type material bis(2(2′-hydroxyphenyl)pyridine)beryllium (Bepp_2_) was used as the interlayer, obtaining a long lifetime. Figure 20a depicts the WOLED architecture: ITO/MeO-TPD: F4-TCNQ (100 nm, 4%)/NPB (20 nm)/2-methyl-9,10-di(2-naphthyl)anthracene (MADN): p-bis(p-*N*,*N*-di-phenyl-aminostyryl) (DSA-ph, 20 nm, 7%)/interlayers (0 or 3 nm)/bis(10-hydroxybenzo[h]quinolinato)beryllium complex (Bebq_2_): Ir(MDQ)_2_(acac), 9 nm, 5%)/Bebq_2_ (25 nm)/LiF/Al, where the interlayers correspond to none, Alq_3_, bis(2-methyl-8-quinolinato)-4-phenylphenolate aluminum (BAlq), Bebq_2_, TPBi and Bepp_2_, respectively. The influence of these n-type interlayers can be briefly summarized. (1) Bebq_2_ was the host for phosphor Ir(MDQ)_2_(acac), which confined triplets within EMLs due to its higher T_1_ (2.25 eV); (2) Structural heterogeneity at the EML/ETL interface was eliminated since Bebq_2_ was also the ETL, improving the lifetime as well as efficiency; (3) 3-nm interlayers can prohibit the Dexter transfer. As a result, only the n-type materials with high T_1_ (TPBi (2.74 eV), Bepp_2_ (2.6 eV)) are effective to ensure white emissions, since they prevent mutual exciton quenching between the fluorophore and phosphor. Particularly, as displayed in Figure 20b, the hybrid WOLED with the Bepp_2_ interlayer shows a lifetime as long as >30,000 h at 1000 cd m^−2^.

#### 6.2.3. Interlayer-Free Hybrid WOLEDs

**Figure 21:**
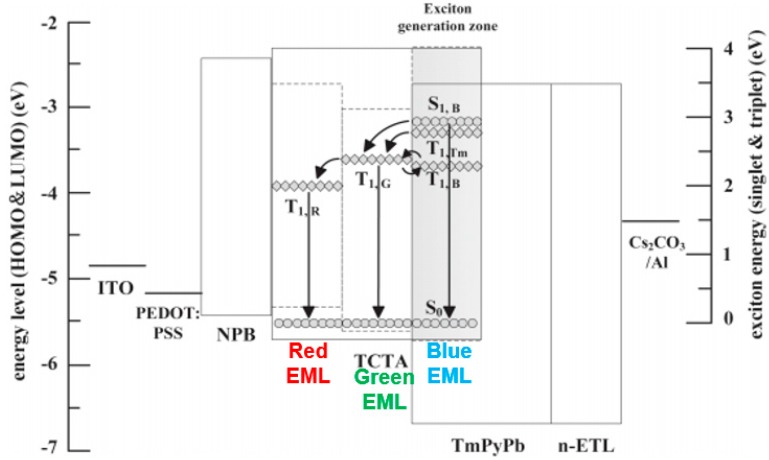
The device structure, energy levels, and EL processes. The main exciton generation zone was marked by gray color. m is TmPyPB. Reproduced from Reference [201].

Avoiding the use of interlayers is possible to simplify low-T_1_ fluorophore-based hybrid WOLEDs, which is more desirable for practical applications. However, it still remains a big challenge [199,200]. In 2004, a novel strategy to loosen the bottleneck was reported by Sun et al. [201]. In their devices, although the blue fluorophore *N*,*N*′-di-1-naphthalenyl-*N*,*N*′-diphenyl-[1,1′:4′,1′′:4′′,1′′′-quaterphenyl]-4,4′′′-diamine (4P-NPD) exhibited a lower T_1_ (2.3 eV) compared with the phosphorescent emitter Ir(ppy)_2_(acac) (2.4 eV), no interlayer was required between the fluorescent and phosphorescent EMLs. This was mainly because the fluorescent EML was constructed by introducing bipolar mixed hosts. As a result, the peak forward-viewing PE of 41.7 lm W^−1^ was achieved, slightly decreased to 34.3 lm W^−1^ at 1000 cd m^−2^, with a CRI as high as 82. Figure 21 depicts the EML structure: TCTA: 4% Ir(MDQ)_2_(acac) (3.5 nm)/TCTA: 8% Ir(ppy)_2_(acac), 5 nm)/TCTA: TmPyPB: (4P-NPD, 73%: 25%: 2%, 7 nm). The working mechanism can be briefly summarized as follows: (1) The blue emission was produced since the singlet excitons were almost all harvested by 4P-NPD; (2) Given that the peak EQE was as high as 19%, red and green emission were strengthened since there was almost no triplet excitons decay non-radiatively onto the blue fluorophoree 4P-NPD; (3) Red and green emission were further enhanced via direct exciton formation onto guests Ir(ppy)_2_(acac) and Ir(MDQ)_2_(acac); (4) The exciton generation zone was widened due to the bipolar mixed hosts, which could reduce the efficiency roll-off.

### 6.3. High-T_1_ Fluorophores Based Hybrid WOLEDs

#### 6.3.1. Basic Concept of High-T_1_ Blue Fluorophore-Based Hybrid WOLEDs

As mentioned above, avoiding the use of an interlayer can make the device structure of hybrid WOLEDs more simplified. To this end, the introduction high-T_1_ blue fluorophores is a very effective strategy. For high-T_1_ blue fluorophore-based hybrid WOLEDs, aside from considering the effect of the interlayer, all generated excitons can be harvested, ensuring the high exciton-harvesting efficiency. In terms of multi-EML structures, a key feature is that phosphorescent emitters can efficiently harness triplet excitons generated in the blue region since singlet and triplet excitons possess different diffusion lengths [202,203,204]. Thus, a 100% IQE could be achieved.

#### 6.3.2. Triplet-Harvesting Structure

**Figure 22:**
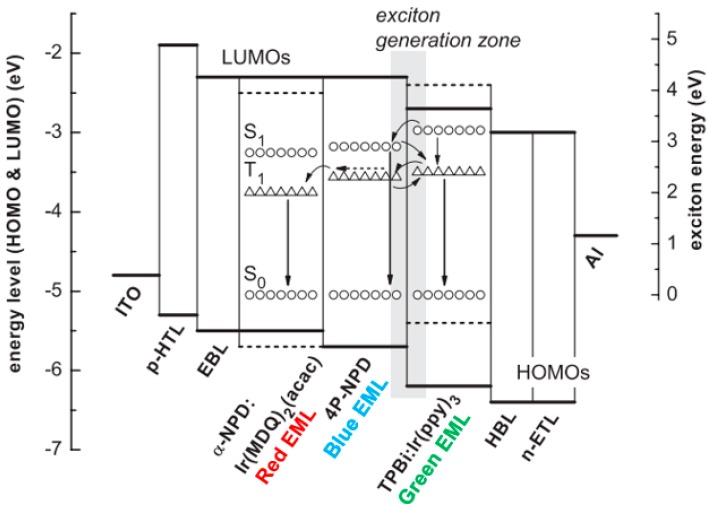
The WOLED structure, energy levels, and EL procedures. Reproduced from Reference [205].

Schwartz et al. designed a triplet-harvesting structure to realize hybrid WOLEDs, which exhibited a total PE of 57.6 at 100 cd m^−2^ and 37.5 lm W^−1^ at 1000 cd m^−2^ [205]. Figure 22 depicts the EML structure: *N*,*N*’-di(naphthalen-1-yl)-*N*,*N*’-diphenyl-benzidine (α-NPD): Ir(MDQ)_2_(acac)/4P-NPD/TPBi: Ir(ppy)_3_. The emission mechanism can be summarized as follows.

(1) Due to different transporting properties (e.g., the hole transporting property of α-NPD and 4P-NPD, as well as the electron transporting property of TPBi), the exciton generation zone is located near the 4P-NPD/TPBi: Ir(ppy)_3_ interface; (2) Ir(ppy)_3_ can harvest all the excitons generated on the TPBi: Ir(ppy)_3_ EML for green emission; (3) Singlet excitons are either harvested by 4P-NPD for blue emission or transferred to the green EML to further enhance the green intensity by dint of the Förster transfer; (4) Due to high T_1_ of 4P-NPD (2.3 eV), triplet excitons can diffuse into the adjacent α-NPD: Ir(MDQ)_2_(acac) EML to be harvested by Ir(MDQ)_2_(acac) for orange emission. Therefore, a very efficient hybrid WOLED was obtained.

#### 6.3.3. Double Blue EMLs Structure

**Figure 23:**
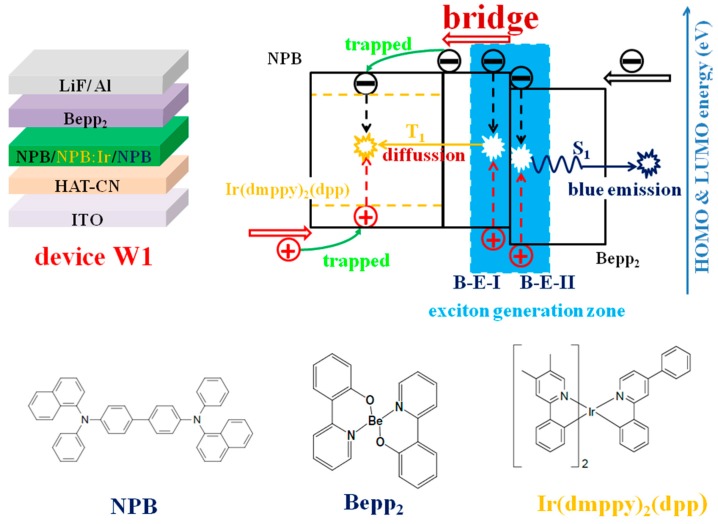
The WOLED architecture, emission mechanism, and emitters chemical structures. Reproduced from Reference [206].

To simplify the device structure and achieve high performance, Liu et al. introduced the double blue EMLs to develop three-layer hybrid WOLEDs, obtaining a total efficiency of 89.3 lm W^−1^ at 100 cd m^−2^ and 65.1 lm W^−1^ at 1000 cd m^−2^ [206]. Moreover, very low voltages were achieved (e.g., 2.4 V at 1 cd m^−2^). Figure 23 depicts the device structure: ITO/HAT-CN (100 nm)/NPB (20 nm)/NPB: Ir(dmppy)_2_(dpp) (35 nm, 15%)/NPB (4.5 nm)/Bepp_2_ (35 nm)/LiF/Al. A key factor for the high performance was using double multifunctional blue EMLs. The 4.5 nm NPB blue EML (B-E-I) is bridge-like, since an amount of electrons can readily go across it to the orange region. Afterwards, these electrons could meet holes to form excitons on phosphorescent guest sites by dint of the charge trapping effect. Thus, the charges and excitons distribution is effectively broadened. On the other hand, the Bepp_2_ blue EML (B-E-II) was employed as the ETL, leading to reduced heterojunction at the EML/ETL interface. The emission mechanisms can be summarized as follows. (1) The main exciton generation zone was situated near NPB/Bepp_2_ interface via the use of p-type material NPB and n-type material Bepp_2_; (2) The generated singlet excitons could be harnessed by NPB or Bepp_2_ for blue emission; (3) Unutilized triplet excitons could be diffused into phosphorescent EML and harnessed for orange emission by Ir(dmppy)_2_(dpp); (4) B-E-I could confine electrons at the NPB/Bepp_2_ interface, guaranteeing the location of the main exciton generation.

#### 6.3.4. Single-EML Hybrid WOLEDs

Compared with multi-EML hybrid WOLEDs, single-EML hybrid WOLEDs possess some unique merits, including a simple architecture, simplified fabricating procedure, reduced heterojunction, and decreased cost [207,208,209,210]. Therefore, the use of a single-EML structure is an effective strategy to achieve high-performance hybrid WOLEDs. One of the most inspiring single-EML hybrid WOLEDs was realized by Peng et al. in 2011, as their device showed a maximum EQE as high as 27.8% [207]. To develop high-performance single-EML hybrid WOLEDs, a key feature is selecting materials that can simultaneously act as hosts for phosphorescent emitters and fluorophores for furnishing blue emissions. As a result, the T_1_ of the selected materials should be higher than that of phosphors, leading to the triplet excitons on the selected materials being effectively harnessed by phosphors rather than decaying non-radiatively.

**Figure 24:**
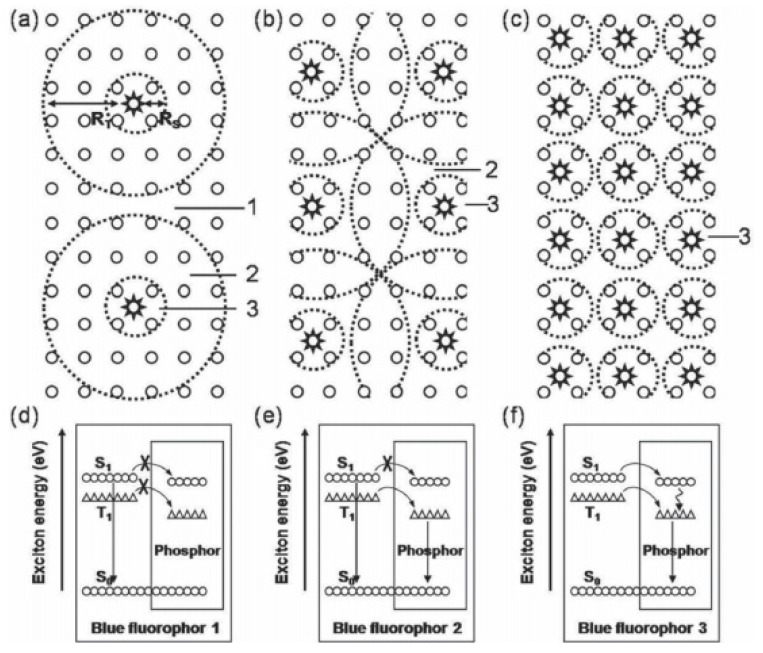
Decay channels for singlets and triplets on the host fluorescent molecules with the changing guest concentration. Reproduced from Reference [211].

By doping 0.1% tris(2-phenylquinoline)iridium(III) (Ir(2-phq)_3_) into the host 2,8-di[4-(diphenylamino)phenyl] dibenzothiophene-S,S-dioxide (DADBT), an efficient single-EML hybrid WOLED was reported by Ye et al. in 2012 [211]. In their device, a maximum total EQE of 26.6% could be achieved. The WOLED architecture is ITO/NPB (30 nm)/TCTA (10 nm)/0.1 wt % Ir(2-phq)_3_: DADBT (30 nm)/TPBI (30 nm)/LiF/Al. The emission mechanisms can be summarized as follows. (1) Due to the bipolar property of DADBT and low concentration of Ir(2-phq)_3_, excitons were formed in whole EML and distributed onto the fluorescent molecules; (2) As displayed in Figure 24a, because singlet excitons have a much shorter diffusion length (~3 nm) than triplet excitons (~100 nm), excitons would decay according to the different guest concentration; (3) As displayed in Figure 24b,e, singlet excitons were harvested by the fluorophore host to produce blue light while triplets could be transferred to phosphor to give off complementary light, if the guest concentration was low enough (e.g., 0.1%). In this situation, a part of host fluorescent molecules could not meet the phosphorescent molecules in the neighborhood (3 nm), but phosphorescent molecules were placed at 100 nm. As a result, singlets and triplets decayed separately, leading to warm-white emission and 100% IQE. In particular, as displayed in Figure 24d, if too low guest concentrations were used, fluorescent molecules could not meet the phosphorescent molecules in their neighborhood (100 nm), and only blue emission would be obtained since triplet excitons would non-radiatively decay. Also, as displayed in Figure 24c,f, if a high guest concentration was utilized (e.g., 10%), phosphorescent molecules would heavily surround the host fluorescent molecules in the 3-nm areas, leading to both singlets and triplets being consumed by the phosphor. Hence, only phosphorescent emissions could be produced.

#### 6.3.5. TADF Hybrid WOLEDs

**Figure 25:**
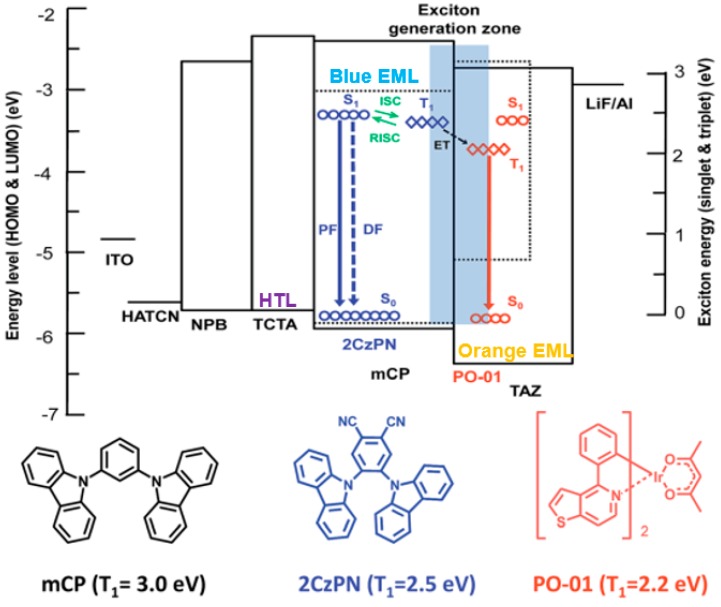
The function mechanism. PF and DF denote prompt and delayed fluorescence, respectively. ET is energy transfer. Reproduced from Reference [215] with permission from the Royal Society of Chemistry.

Blue TADF emitters usually exhibit high T_1_, which can be introduced to construct high-performance hybrid WOLEDs [212,213,214]. In 2014, a TADF hybrid WOLED was firstly developed by Zhang et al., obtaining a peak efficiency as high as 47.6 lm W^−1^ [215]. Figure 25 depicts the WOLED architecture: ITO/HATCN (5 nm)/NPB (40 nm)/TCTA (10 nm)/mCP: 4,5-bis(carbazol-9-yl)-1,2-dicyanobenzene (2CzPN, 11 nm, blue EML)/TAZ: 4 wt % PO-01 (4 nm, orange EML)/TAZ (40 nm)/LiF/Al. The high performance can be explained as follows. (1) Since mCP possesses a large energy gap as well as a T_1_ as high as 3.0 eV, it was selected as a host of the blue TADF emitter 2CzPN; (2) 2CzPN was situated closest the exciton generation area, leading to excitons being diffused into the whole EML for balanced emissions; (3) Triplet excitons generated onto the blue TADF emitter were harnessed via Dexter transfer to the phosphorescent emitter or by the RISC process to the emitting singlet state; (4) By dint of the charge trapping effect of 2CzPN, the recombination zone was stable with increasing voltages, obtaining an excellent color-stability.

#### 6.3.6. p-Type Delayed Fluorescence Hybrid WOLEDs

To prevent T_1_ loss, the trapped T_1_ should be recycled in hybrid WOLEDs in order to achieve high performance. Different from the TADF mechanism, another possible triplet up-conversion mechanism is TTA or p-type delayed fluorescence. Based on such a strategy, hybrid WOLEDs also show the potential to achieve high performance. Since such a kind of hybrid WOLED is unlike low-T_1_ blue fluorophore-based hybrid WOLEDs, in which the fluorophores cannot harvest triplets, we classified p-type delayed fluorescence hybrid WOLEDs in this section.

**Figure 26:**
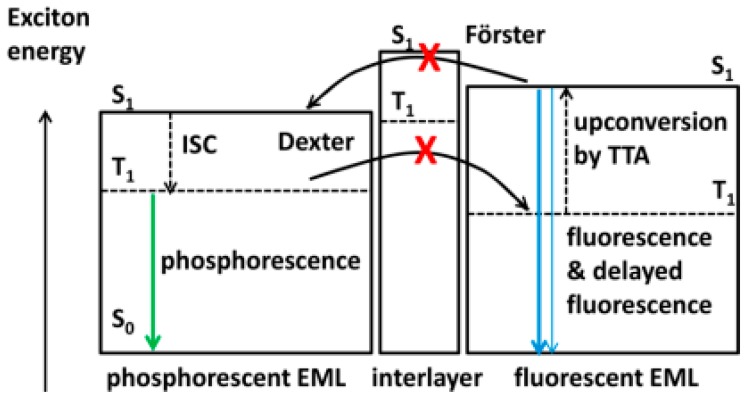
Exciton energy diagram of the WOLEDs and the possible methods of decay of singlet and triplet excitons. Reprinted with permission from Reference [216]. Copyright (2016) American Chemical Society.

Zhang et al. used this strategy, obtaining a maximum EQE of 19.1%, PE of 49.3 lm W^−1^, together with a CRI of 88 and a lifetime of over 10,000 h at 1000 cd m^−2^ [216]. The WOLED architecture is as follows: ITO/96% 2-TNATA: 4% F4TCNQ (150 nm)/BPAPF (20 nm)/97% BPAPF: 3% Ir(MDQ)_2_acac (15 nm)/42.5% BPAPF: 42.5% SBFK: 15% Irppy_3_ (10 nm)/50% BPAPF: 50% SBFK (5 nm)/95% α,β-ADN: 5% DACrs (30 nm)/9,10-bis(4-(2-phenyl-1Hbenzo[d]imidazol-1-yl)phenyl)anthracene (BPBiPA, 15 nm)/LiF/Al, where BPAPF is 9,9-bis(4-(di(p-biphenyl)-aminophenyl)) fluorene, SBFK is bis(9,9′-spirobifluorene-2-yl) ketone and DACrs is 6,12-bis(di(3,4-dimethylphenyl)amino)chrysene. The high performance can be explained as follows. (1) The mixed host of green phosphorescent EML as well as the spacer could enlarge the recombination zone and reduce the triplet concentration near the blue fluorophores; (2) Blue fluorophore with TTA was adopted to recycle the trapped T_1_; (3) An electron transport material with both high electron mobility and good exciton confinement ability, namely, BPBiPA, was used to boost the TTA efficiency. Consequently, the trapped triplets by the blue fluorophores were greatly reduced. What is more, once being trapped, the triplet excitons could also be recycled by the TTA process, as can be seen from Figure 26.

## 7. Doping-Free WOLEDs

Compared with doping technologies, the doping-free technique possesses many advantages, such as avoiding hosts, simplifying device architectures, shortening fabrication procedures, and reducing the cost. Therefore, doping-free WOLEDs have great potential for practical applications [217,218,219,220,221,222,223,224,225]. So far, although it is still challenging to achieve high-performance doping-free WOLEDs, some strategies have been reported to alleviate these difficulties.

**Figure 27:**
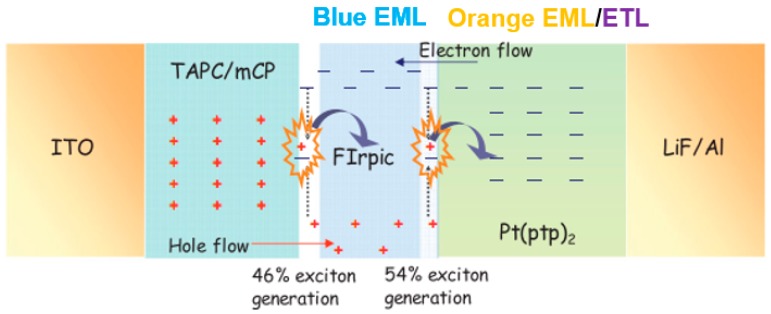
Proposed operational principles of the doping-free WOLED. Reproduced from Reference [226].

Wang et al. described a doping-free phosphorescent WOLED via the use of a neat metal-organic Pt(II)-pyridylazolate phosphor bis[3,5-bis(2-pyridyl)-1,2,4-triazolato]platinum(II) [Pt(ptp)_2_] possessing a nearly unity quantum yield and excellent electron-transporting ability, achieving a PE of approximately 50 lm W^−1^ [226]. Their device has the architecture of ITO/TAPC/mCP (10 nm)/FIrpic (8 nm)/Pt(ptp)_2_ (90 nm)/LiF/Al. The reason for the high PE can be explained by the following. (i) Eliminating host materials that possess high energy gaps allows a direct charge recombination on the molecular sites of phosphorescent dyes, which reduces the host-guest exchange energy loss in PE; (ii) the high quantum yield of neat Pt(ptp)_2_ assures that the electrically generated excitons can be utilized efficiently by radiative decay; (iii) using a single Pt(ptp)_2_ layer as a homogeneous EML/ETL is one determining factor for voltage reduction, which can enhance the PE. The operational principles of the doping-free WOLED are described in Figure 27. In this device, FIrpic was used as an an ambipolar material. Therefore, excitons could be generated on both edges of the FIrpic layer (46% vs. 54%), and then harvested by FIrpic and Pt(ptp)_2_ molecules, respectively, in an independent manner.

**Figure 28:**
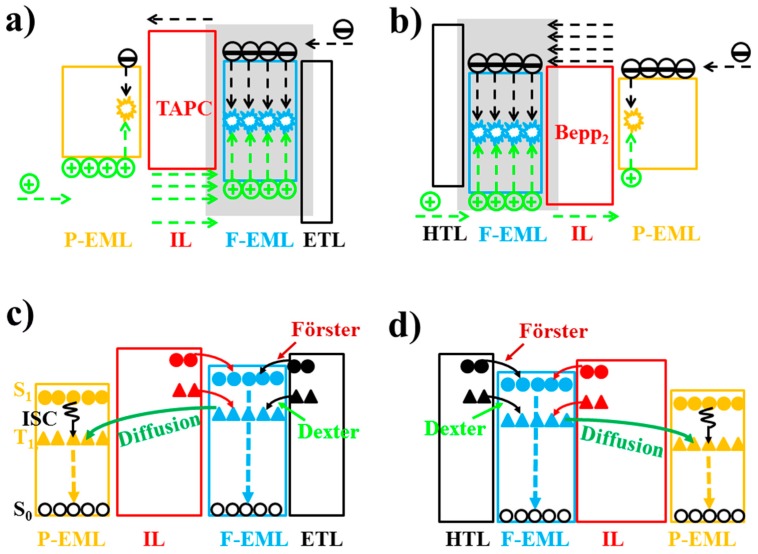
Diagrams of working mechanisms for different structures. The charge distribution for (**a**) devices W21 and (**b**) W22. The exciton distribution for devices (**c**) W21 and (**d**) W22. Reproduced from Reference [227].

The doping-free technique can not only be applied to achieve phosphorescent WOLEDs, but also be used to realize high-performance hybrid WOLEDs. By employing <1 nm EMLs, high-performance doping-free hybrid WOLEDs were achieved by Liu et al. [227]. Low voltage, bright luminance, good color-stability, as well as excellent PE (~40 lm W^−1^) could be obtained in their two-color device. Besides, the CCT range could be as broad as 2325–8011 K and the CRI was as high as 91.3 in their three-color device. Two-color devices have the device structure of ITO/HAT-CN (100 nm)/NPB (15 nm)/TAPC (5 nm)/EMLs/TmPyPB (35 nm)/LiF/Al, in which the EMLs were formed by Ir(dmppy)_2_(dpp) (0.9 nm)/TAPC (3.5 nm)/DSA-ph (0.5 nm) and DSA-ph (0.5 nm)/Bepp_2_ (3.5 nm)/Ir(dmppy)_2_(dpp) (0.9 nm) for devices W21 and W22, respectively. The working mechanism of device W21 is explained below. (1) As displayed in Figure 28a, excitons were mainly formed on DSA-ph as well as DSAph/TmPyPB and TAPC/DSAph interfaces; (2) Blue emission was produced by singlet excitons directly recombined on DSA-ph; (3) Blue emission was further increased due to singlet excitons transferred from TAPC and TmPyPB by the Förster process; (4) Yellow emission was generated by excitons recombined in the phosphorescent EML, since some electrons could pass though this 3.5-nm interlayer TAPC to meet holes; (5) Yellow emission was further enhanced since triplet excitons diffused from the exciton formation zone were harnessed by the phosphor (Figure 28c). The working mechanism of device W22 is explained below. (1) As displayed in Figure 28b, excitons were mainly formed on DSA-ph as well as DSA-ph/Bepp_2_ and TAPC/DSAph interfaces; (2) Blue emission was not only produced by singlet excitons directly recombined on DSA-ph, but also increased due to singlets transferred from TAPC and Bepp_2_; (3) Yellow emission was not only generated by excitons recombined in the phosphorescent EML, since some holes could pass though this 3.5-nm interlayer Bepp_2_ to meet electrons, but also enhanced since triplet excitons diffused from the exciton formation zone were harnessed by the phosphor (Figure 28d).

**Figure 29:**
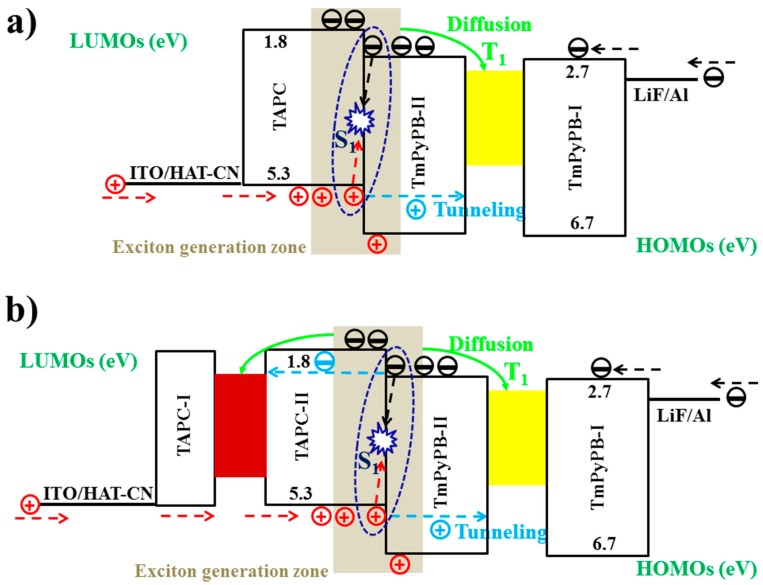
Schematic illustration of the emission mechanisms of (**a**) single-molecular-emitter BMEF/DFH-WOLEDs; (**b**) two-molecular-emitter BMEF/DFH-WOLEDs. Reprinted with permission from Reference [228]. Copyright (2017) American Chemical Society.

In general, blue molecular emitters are the key to the development of hybrid WOLEDs. To avoid considering the effect of blue molecular emitters in addition to taking advantage of the doping-free technology, it is beneficial to achieve blue molecular emitter-free and doping-free hybrid WOLEDs (BMEF/DFH-WOLEDs). Luo et al. developed BMEF/DFH-WOLEDs WOLEDs via the manipulation of exciplex and electroplex emissions [228]. For the BMEF/DFH-WOLED with a single yellow molecular emitter, it exhibited the maximum total EQE and PE of 16.8% and 56.4 lm W^−1^, respectively. At 1000 cd m^−2^, the PE was still as high as 40.0 lm W^−1^. For the two-molecular-emitter BMEF/DFH-WOLED, it could exhibit a CRI of 92.1, a CCT of 2319 K, an EQE of 15.1%, and a PE of 28.2 lm W^−1^. Single-molecular-emitter WOLEDs have the structure of ITO/HATCN/TAPC (25 nm)/TmPyPB (10 nm)/Ir(dmppy)_2_(dpp) (0.3 nm)/TmPyPB/Cs_2_CO_3_/Al. Two-molecular-emitter WOLEDs have the structure of ITO/HATCN (100 nm)/TAPC (22 nm)/Ir(piq)_3_ (0.3 nm)/TAPC (3 nm)/TmPyPB (20 nm)/Ir(dmppy)_2_(dpp) (0.3 nm)/TmPyPB (35 nm)/Cs_2_CO_3_/Al. The emission mechanisms can be explained as follows. As shown in Figure 29a, for single-molecular-emitter WOLEDs, the blue emission originated from the exciplex/electroplex system, the yellow emission resulted from (i) the exciplex/electroplex system, since the exciplex/electroplex emission occurs at all luminances, although it is hard to distinguish its emission from Ir(dmppy)_2_(dpp); (ii) triplets formed on TAPC, TmPyPB as well as the exciplex/electroplex system via the diffusion process; and (iii) singlet and triplet excitons directly formed on Ir(dmppy)_2_(dpp) due to the tunneling effect. As shown in Figure 29b, for two-molecular-emitter WOLEDs, the blue and yellow emission mechanisms are almost the same as those of single-molecular-emitter WOLEDs. However, the amount of excitons harvested for the blue and yellow emissions is less, since some excitons were harvested by Ir(piq)_3_ for red emission. On the other hand, for the red emission, as Ir(piq)_3_ is closer to the exciton generation zone than Ir(dmppy)_2_(dpp), both singlets and triplets in the exciton generation zone were easily harvested by Ir(piq)_3_.

**Figure 30:**
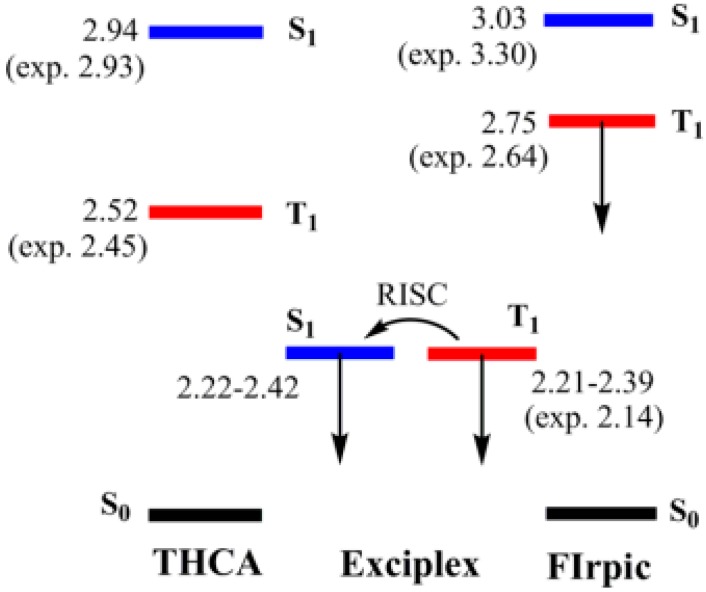
Energy diagram for the S_1_ and T_1_ states of THCA, FIrpic, and their mixture (THCA/FIrpic), which is responsible for the exciplex emission. Reprinted with permission from Reference [235]. Copyright (2015) American Chemical Society.

Exciplexes have small exchange energies since the compact electron densities from the LUMO and HOMO of the charge-transfer complex are separated by a relatively long distance [229,230,231,232,233,234]. Due to the broad band of exciplex emission, the mixing of phosphorescent and exciplex emissions is another strategy to achieve high-performance WOLEDs. Cherpak et al. developed a unique doping-free WOLED by combining the blue emission from FIrpic (470 nm) and a broad delayed fluorescence induced by thermal activation (i.e., TADF emission, 570 nm) with additional direct phosphorescence from the triplet exciplex (635 nm) formed at the star-shaped hole transporting compound tri(9-hexylcarbazol-3-yl)-amine (THCA) and FIrpic interface, achiveing a maximum EQE of 5%, luminance of 38,000 cd m^−2^, and low voltages (e.g., 2 cd m^−2^ at 2.3 V) [235]. The device architecture is ITO/CuI (8 nm)/THCA (40 nm)/FIrpic(9 nm)/3,6-di(9-carbazolyl)-9-(2-ethylhexyl)carbazole (TCz1, 10 nm)/Ca (50 nm)/Al (200 nm). As shown Figure 30, there are high-energy barriers for electron (0.92 eV) and for hole (1.17 eV) transfer between THCA and FIrpic. Hence, the exciplex formation by the two compouds is possible, where THCA is an electron donor and FIrpic serves as an electron acceptor. For the exciplex, both S_1_ and T_1_ states of the THCA/FIrpic aggregates are quasi-degenerate with a very small positive S_1_-T_1_ splitting, leading to the fact that the T_1_ state can be populated by the RISC process through a thermally activated up-conversion mechanism at room temperature. In other words, the S_1_ state of the exciplex could be populated by the thermal activation from the triplet exciplex produced by electron-hole recombination in the EML with a 3:1 ratio (triplets/singlets), achieving the TADF emission. Therefore, an up-conversion from the nonradiative triplet state to the radiative singlet state due to the exciplex formation between THCA and FIrpic is achieved. Besides, the triplet exciplex provides its own radiative activity because of the large spin-orbit coupling contribution at the metal center. Thus, the wide EL spectrum is obtained; both states provide emissions with slightly different lifetimes.

## 8. Summary and Outlook

In this review, the strategies for achieving high-performance WOLEDs are described. However, it must be pointed out that the presented state-of-the-art strategies can be also helpful to achieve other kinds of LEDs (e.g., polymer LEDs, quantum-dot LEDs, nanoplatelet LEDs, and perovskite LEDs) [236,237,238,239,240,241,242,243,244,245] and related optoelectrical devices [246,247,248]. Besides, since luminescence is a type of cold body radiation caused by external stimuli (e.g., electric field), the strategies, particularly for the outcoupling strategies, are also useful to obtain luminescence from other types of external stimuli (e.g., mechanical stress, photoabsorption, and chemical reactions) [249,250,251,252,253,254,255,256]. The chemical structures of organic compounds as the emitters, hosts, and other part of WOLEDs are presented in Appendix A (Table A1). The representative device structures and their detailed performance at each class of WOLEDs are shown in Table 1.

Despite the fact that the performance of WOLEDs has been enhanced via various effective strategies, some challenges still need to be overcome before the wholesale commercialized productions are realized, including maximizing efficiency, lifetime, and as cost. Since the theoretical limitation for the efficiency is 248 lm W^−1^, more efforts are required to further enhance the performance [257]. In general, the PLQY of emitters, charge balance, as well as the outcoupling factor are the key to determining the efficiency. As a result, the selection of excellent materials, the careful manipulation of charges and excitons distribution, and the utilization of outcoupling techniques are crucial [258,259,260,261,262].

To effectively enhance the lifetime, an alternative strategy is developing tandem WOLEDs. In addition, the introduction of advanced encapsulation technology is another effective strategy to achieve long lifetime. This is because both oxygen and moisture are detrimental to the stability of OLEDs. Without encapsulation, OLEDs can degrade easily. Hence, to obtain the ideal encapsulating barriers (10^−6^ g/m^2^/day), encapsulation technology should also be enhanced.

So far, the high cost is one of the main obstructions for the mass production of OLEDs. To meet the requirement of consumers, the price needs to be under 20 $/m^2^. Besides, OLED displays are more expensive (>150%) than the mainstream LCDs. To reduce the cost, the solution-processed technique is conducive, although the performance of solution-processed WOLEDs is still not high enough. Additionally, doping-free WOLEDs are promising to lower the cost, since they naturally exhibit simple characteristics. Moreover, new fabrication ways, simplified architectures, and excellent yet cheap materials deserve to be considered. With continue efforts from both industrial and academic researchers, the performance of WOLEDs is believed to be high enough to satisfy the demand of consumers in the near future.

## Figures and Tables

**Table 1:** Summary of the performances of WOLEDs with representative device structures.

Devices ^a^	V_on_/V_1000_^b^ (v)	EQE_max_/EQE_1000_^c^ (%)	CE_max_/CE_1000_^d^ (cd A^−1^)	PE_max_/PE_1000_^e^ (lm W^−1^)	CIE_1000_^f^	CRI ^g^
Ref. [120] ^h^	3.0/-	5.6/-	14.0/-	9.2/-	(0.334, 0.337)	81
Ref. [123] ^h^	-/-	6.3/-	14.7/-	-/-	(0.335, 0.355)	-
Ref. [124] ^i^	3.3/5	12/-	-/-	42/-	(0.43, 0.45)	80
Ref. [125] ^i^	-/-	19.3/-	-/-	72.2/-	(0.33, 0.39)	71
Ref. [132] ^i^	-/-	-/-	113.6/111.7	92.5/75.5	(0.288, 0.485)	-
Ref. [12] ^i^	2.5/3.6	28.1/21.5	83.6/61.0	105/59.5	(0.40, 0.48)	-
Ref. [133] ^i^	2.83/3.79	-/25	-/-	/44	(0.335, 0.396)	68
Ref. [134] ^i^	-/3	-/34	-/-	-/90	(0.41, 0.49)	69
Ref. [135] ^i^	-/-	20.1/-	-/-	41.3/-	(0.38, 0.45)	85
Ref. [136] ^i^	-/-	-/24.5	-/57.7	-/33.8	(0.44, 0.46)	81
Ref. [9] ^i^	-/<4	56.1/54.6	-/142	132.8/123.4	(0.339, 0.458)	-
Ref. [156] ^j^	3.6/7.8	17/8.5	45.6/20.6	34.1/8.3	(0.33, 0.41)	-
Ref. [160] ^j^	3.4/5.4	19.2/14.2	51.4/37.3	47.5/21.8	(0.348, 0.457)	69
Ref. [184] ^k^	-/-	18.7/-	-/-	37.6/-	(0.40, 0.41)	85
Ref. [193] ^k^	-/-	-/-	70.2/-	43.4/-	(0.44, 0.50)	46
Ref. [198] ^k^	2.8/-	-/-	20.6/19.0	20.9/11.9	(0.277, 0.354)	73
Ref. [201] ^k^	3.1/-	32.3/28.9	76.8/68.9	70.9/58.3	(0.43, 0.43)	83
Ref. [205] ^k^	~2.5/3.46	-/16.1	-/-	-/37.5	(0.44, 0.47)	86
Ref. [206] ^k^	2.4/3.1	-/-	84.5/-	106.3/65.1	(0.43, 0.48)	47
Ref. [211] ^k^	2.4/-	26.6/21.2	53.5/42.6	67.2/33.5	(0.46, 0.44)	-
Ref. [215] ^k^	-/-	38.4/26.2	-/-	80.1/37.5	(0.45, 0.48)	-
Ref. [216] ^k^	-/-	19.1/17.6	49.6/-	49.3/-	(0.43, 0.46)	80
Ref. [226] ^l^	2.4/-	-/-	-/-	49.5/30.0	(0.44, 0.47)	65
Ref. [227] ^l^	2.5/-	11.6/-	32.5/-	39.3/18.2	(0.42, 0.48)	-
Ref. [228] ^l^	-/-	16.8/-	49.6/-	56.4/40.0	(0.42, 0.51)	56
Ref. [235] ^l^	2.2/-	5/-	15/-	-/-	-	-

^a^ The structures are mentioned before. ^b^ The turn-on voltage (1 cd/m^2^) and the voltage at 1000 cd/m^2^. ^c^ Maximum total EQE and the EQE at 1000 cd/m^2^. ^d^ Maximum total CE and the CE at 1000 cd/m^2^. ^e^ Maximum total PE and the PE at 1000 cd/m^2^. ^f^ CIE coordinates at about 1000 cd/m^2^. ^g^ Maximum CRI. ^h^ Fluorescent WOLEDs. ^i^ Phosphorescent WOLEDs. ^j^ TADF WOLEDs. ^k^ Hybrid WOLEDs. ^l^ Doping-free WOLEDs.

## Appendix A

**Table A1:** Chemical structures of organic compounds as the emitters, hosts, and other part of WOLEDs discussed.

Names	Chemical Structures	Functions
2-TNATA		HIL
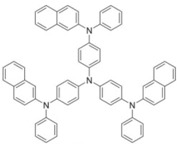
3CzTRZ		Host
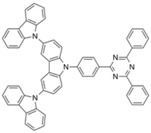
3DTAPBP		HIL
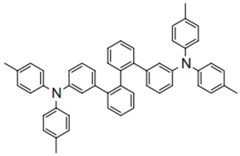
4CzPN		Green TADF emitter
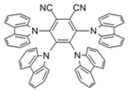
4CzTPN-Ph		Red TADF emitter
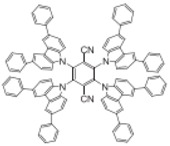
4P-NPD		Host, HTL
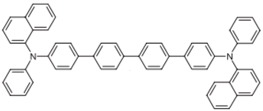
α-NPD		Host, HTL
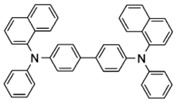
Alq_3_		Green fluorophore, host, ETL
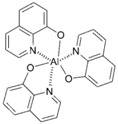
ADN		Host
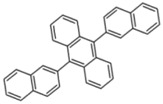
α,β-AND		Host
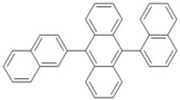
BAlq		ETL
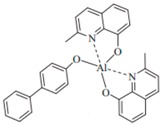
B3PyMPM		ETL
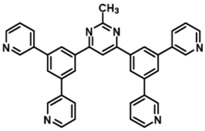
B4PyMPM		ETL
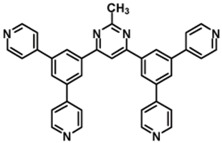
BCzVBi		Blue fluorophore
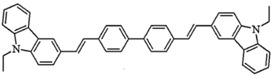
BCP		Host, ETL
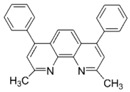
Bebq_2_		ETL
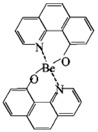
Bepp_2_		Blue fluorophore, Host, ETL
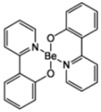
BPBiPA		ETL
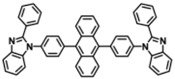
Bphen		Host, ETL
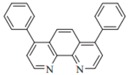
BTPEAn		Blue fluorophore
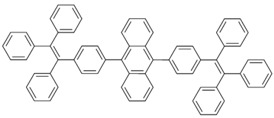
CBP		Host
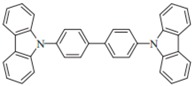
DACrs		Blue fluorophore
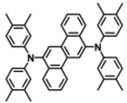
DADBT		Host
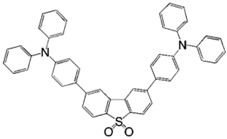
DBP		Red fluorophore
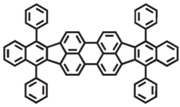
DCM		Orange fluorophore
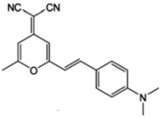
DCzPPy		Host
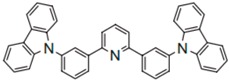
DDAF		Yellow fluorophore
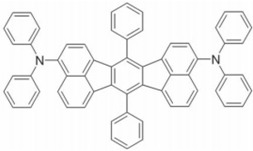
DSA-ph		Blue fluorophore
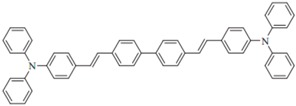
ENPN		Blue fluorophore
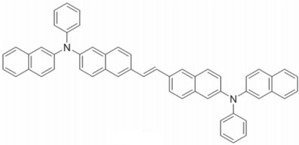
F4-TCNQ		HIL
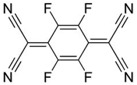
(fbi)_2_Ir(acac)		Orange phosphor
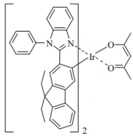
FIr6		Blue phosphor
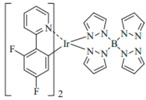
FIrpic		Blue phosphor
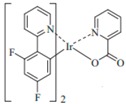
HAT-CN		HIL
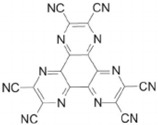
Ir(2-phq)_3_		Orange phosphor
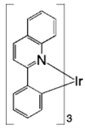
Ir(dmppy)_2_(dpp)		Yellow phosphor
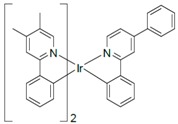
Ir(MDQ)_2_(acac)		Red phosphor
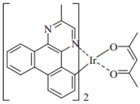
Ir(piq)_3_		Red phosphor
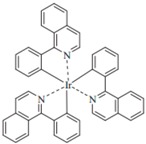
Ir(ppy)_2_(acac)		Green phosphor
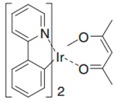
Ir(ppy)_3_		Green phosphor
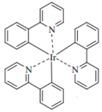
Liq		EIL
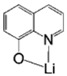
MADN		Host
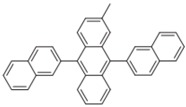
mCP		Host, HTL
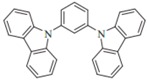
mCBP		Hosr
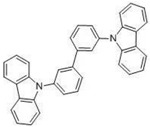
MeO-TPD		HIL
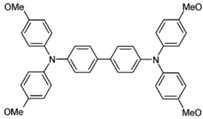
m-MTDATA		HTL
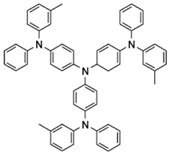
(MPPZ)_2_Ir(acac)		Orange phosphor
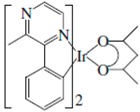
N-1-PhTPA		Blue fluorophore
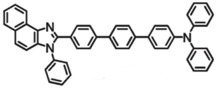
NPB		Host, HTL
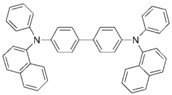
PEDOT: PSS		HIL
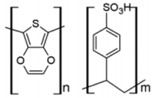
PO-01		Yellow phosphor
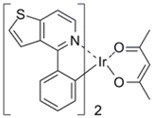
(PPQ)_2_Ir(acac)		Red phosphor
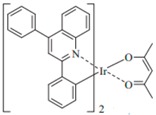
PQIr		Red phosphor
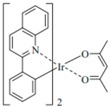
Pt(ptp)_2_		Orange phosphor
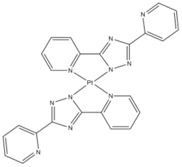
PXZDSO2		Yellow TADF emitter
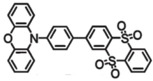
TAPC		HTL
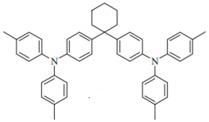
TAZ		ETL
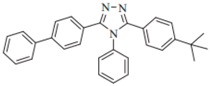
TCTA		Host, HTL
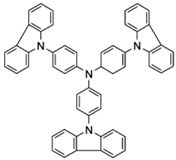
THCA		HTL
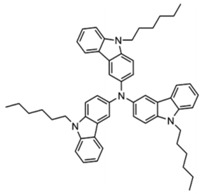
TmPyPB		ETL
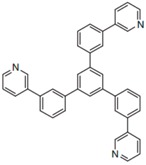
TPBi		Host, ETL
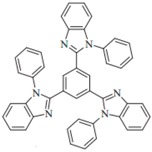
TPD		HTL
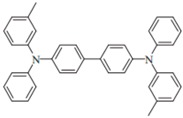
Tris-PCz		HTL
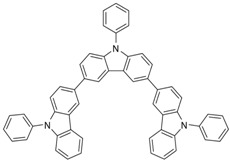
UGH2		Host
